# Blood progenitor redox homeostasis through olfaction-derived systemic GABA in hematopoietic growth control in *Drosophila*

**DOI:** 10.1242/dev.199550

**Published:** 2021-12-01

**Authors:** Manisha Goyal, Ajay Tomar, Sukanya Madhwal, Tina Mukherjee

**Affiliations:** 1 Institute for Stem Cell Science and Regenerative Medicine (inStem), GKVK, Bellary Road, Bangalore 560065, India; 2 The University of Trans-Disciplinary Health Sciences and Technology (TDU), Bengaluru, Karnataka 560064, India; 3 Manipal Academy of Higher Education, Manipal, Karnataka 576104, India

**Keywords:** GABA metabolism, Succinate, Myeloid-progenitor, OXPHOS, Redox-homeostasis, TCA

## Abstract

The role of reactive oxygen species (ROS) in myeloid development is well established. However, its aberrant generation alters hematopoiesis. Thus, a comprehensive understanding of events controlling ROS homeostasis forms the central focus of this study. We show that, in homeostasis, myeloid-like blood progenitor cells of the *Drosophila* larvae, which reside in a specialized hematopoietic organ termed the lymph gland, use TCA to generate ROS. However, excessive ROS production leads to lymph gland growth retardation. Therefore, to moderate blood progenitor ROS, *Drosophila* larvae rely on olfaction and its downstream systemic GABA. GABA internalization and its breakdown into succinate by progenitor cells activates pyruvate dehydrogenase kinase (PDK), which controls inhibitory phosphorylation of pyruvate dehydrogenase (PDH). PDH is the rate-limiting enzyme that connects pyruvate to the TCA cycle and to oxidative phosphorylation. Thus, GABA metabolism via PDK activation maintains TCA activity and blood progenitor ROS homeostasis, and supports normal lymph gland growth. Consequently, animals that fail to smell also fail to sustain TCA activity and ROS homeostasis, which leads to lymph gland growth retardation. Overall, this study describes the requirement of animal odor-sensing and GABA in myeloid ROS regulation and hematopoietic growth control.

## Abstract (precis)

**Summary:** Ablation of olfactory receptor neurons reveals that odor-sensing and GABA are involved in myeloid reactive oxygen species regulation and hematopoietic growth control.

## INTRODUCTION

The use of reactive oxygen species (ROS) as a physiological signal in immune progenitor development is apparent both in vertebrates and invertebrates (Bigarella et al., 2014; Harris et al., 2013; Prieto-Bermejo et al., 2018; Takubo et al., 2013; Tothova et al., 2007; Vincent and Crozatier, 2010). The developmental roles for ROS are reliant on its threshold, as aberrant generation of ROS alters immune progenitor maintenance, differentiation or function (Dragojlovic-Munther and Martinez-Agosto, 2012; Owusu-Ansah and Banerjee, 2009). Thus, mechanisms underlying ROS homeostasis during hematopoiesis are an integral component of redox signaling. In this context, an understanding of metabolic programs that enable immune progenitors to coordinate their ROS levels forms the central focus of our investigation.

*Drosophila* larval blood progenitors akin to the mammalian common myeloid progenitors (CMP) reside in a hematopoietic organ termed the lymph gland. These progenitor cells maintain elevated ROS, the homeostasis of which is necessary for their development. Although physiological ROS sensitize progenitor cells to differentiation cues, their excessive production causes oxidative stress and loss of progenitor homeostasis (Dragojlovic-Munther and Martinez-Agosto, 2012; Owusu-Ansah and Banerjee, 2009). Factors governing progenitor maintenance also include signaling proteins and metabolites emanating from the local niche (posterior signaling center, PSC), differentiating hemocytes, and systemic cues derived from the brain and fat body (reviewed by Banerjee et al., 2019). These include signaling proteins such as Hh (Mandal et al., 2007), wingless (Sinenko et al., 2009), JAK/STAT (Makki et al., 2010), Dpp (Dey et al., 2016), TGFβ (Makhijani et al., 2017) and insulin (Benmimoun et al., 2012), and metabolites such as lipids (Tiwari et al., 2020), adenosine (Mondal et al., 2011), amino acids (Shim et al., 2012) and GABA (Shim et al., 2013; Madhwal et al., 2020). With these intrinsic features of metabolic and signaling requirements, the lymph gland offers a perfect developmental model with which to gain a comprehensive view of programs that control progenitor ROS homeostasis during hematopoiesis.

A key source of ROS in cells is carbon cycling or the tricarboxylic acid (TCA) cycle (Quinlan et al., 2012; Sabharwal and Schumacker, 2014). The TCA cycle generates multiple intermediates that control mitochondrial oxidative phosphorylation (OXPHOS) and lead to ROS generation (Kaplon et al., 2013; Mailloux et al., 2016; Quinlan et al., 2012; Starkov et al., 2004). Through its transport into mitochondria, pyruvate (which is the end-product of glycolysis) functions as a master fuel input driving the TCA cycle and oxidative phosphorylation (Gray et al., 2014; Wang et al., 2016). Pyruvate is converted to acetyl-CoA via pyruvate dehydrogenase (PDH), the key enzyme linking glycolysis to the TCA cycle. While PDH is inactivated by phosphorylation driven by pyruvate dehydrogenase kinase (PDK), the dephosphorylation of PDH by pyruvate dehydrogenase phosphatase (PDP) activates PDH, thereby fueling the TCA cycle (Bowker-Kinley et al., 1998; Harris et al., 2002; Patel et al., 2014).

Our previous work has implicated olfaction and its downstream signaling-mediated release of neuronally derived GABA in progenitor maintenance (Shim et al., 2013) and the immune response (Madhwal et al., 2020). Systemic GABA is sensed by blood progenitors both as a signaling entity and as a metabolite. Use of GABA as a metabolite by progenitor cells and its catabolism to succinate is necessary to mount a successful immune response (Madhwal et al., 2020). In this study, we explore the importance of the GABA metabolic pathway in governing progenitor ROS homeostasis. We find that, in homeostatic conditions, the fueling of the TCA cycle with pyruvate and activation of succinate dehydrogenase (SDH), a key TCA enzyme and component of mitochondrial Complex II, leads to ROS generation in progenitor cells. However, a rise in TCA cycle activity and excessive ROS generation inhibits growth of the lymph gland and leads to loss of progenitor maintenance. Thus, to moderate TCA cycle activity, progenitor cells adopt GABA catabolic pathway to limit the entry of pyruvate into the TCA cycle. Specifically, GABA catabolism into succinate maintains active PDK function. This suppresses PDH enzymatic activity, leading to a lower TCA cycle rate and better control of ROS generation. Finally, we show that animals use environmental odors to moderate TCA cycle activity of progenitors (and consequently redox balance) as a means to coordinate lymph gland growth. Overall, the work presented here describes the use of a systemically derived metabolite in blood progenitor metabolic homeostasis and growth control.

## RESULTS

### GABA metabolism in blood progenitor cells controls the overall size of the lymph gland

**Fig. 1.**
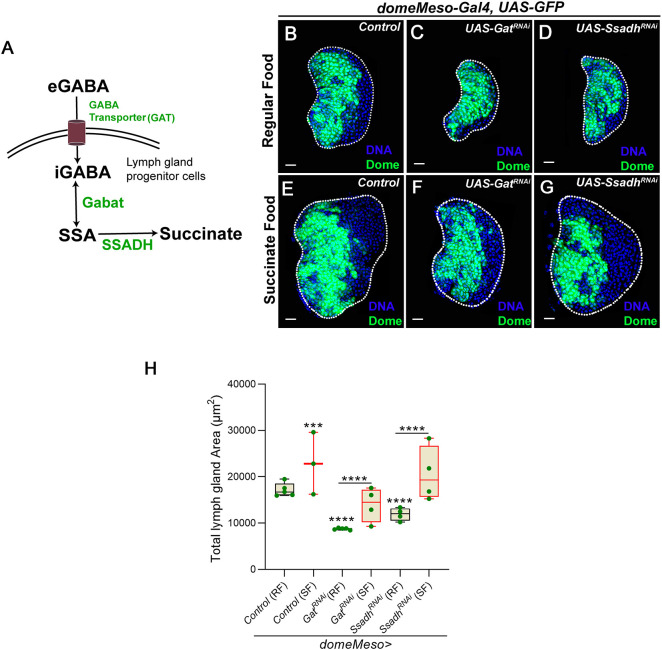
**GABA catabolism in *Drosophila* blood progenitor cells controls lymph gland growth.** (A) Schematic representation of the GABA catabolic pathway. Uptake of extracellular GABA (eGABA) via the GABA transporter (GAT) in blood progenitor cells and its intracellular catabolism (iGABA) into succinic-semialdehyde (SSA) by GABA-transaminase (Gabat) and into the final product, succinate, by succinic semialdehyde dehydrogenase (SSADH), which is the rate limiting step of GABA catabolic pathway. (B-G) Representative images showing lymph gland size. (B) Control (RF, *domeMeso-Gal4,UAS-GFP/+*). (C) *Gat^RNAi^* (RF, *domeMeso-Gal4,UAS-GFP;UAS-Gat^RNAi^*) and (D) *Ssadh^RNAi^* (RF, *domeMeso-Gal4,UAS-GFP;UAS-Ssadh^RNAi^*) produce a reduction in lymph gland size. (E-G) Feeding succinate to (E) control (*domeMeso-Gal4,UAS-GFP/+*), (F) *Gat^RNAi^* (*domeMeso-Gal4,UAS-GFP;UAS-Gat^RNAi^*) and (G) *Ssadh^RNAi^* (*domeMeso-Gal4,UAS-GFP;UAS-Ssadh^RNAi^*) increases lymph gland size when compared with B, C and D, respectively. (H) Quantification of lymph gland area in *domeMeso>GFP/+* (control, RF, *N*=5, *n*=48), *domeMeso>GFP/+* (SF, *N*=3, *n*=36, *P*=0.0005), *domeMeso>GFP/Gat^RNAi^* (RF, *N*=5, *n*=50, *P*<0.0001), *domeMeso>GFP/Gat^RNAi^* (SF, *N*=4, *n*=38, *P*<0.0001), *domeMeso>GFP/Ssadh^RNAi^* (RF, *N*=4, *n*=33, *P*<0.0001) and *domeMeso>GFP/Ssadh^RNAi^* (SF, *N*=4, *n*=32, *P*<0.0001). RF, regular food; SF, succinate-supplemented food. The horizontal line indicates the median; whiskers indicate the minimum and maximum values; box indicates the lower and upper quartiles (***P<0.001, ****P<0.0001, two-way ANOVA, Tukey's multiple comparisons test). Scale bars: 20 µm. *n*, lymph gland lobes; *N*, number of experimental repeats (green dot). DAPI marks DNA. Comparisons for significance are with control values, unless marked by horizontal lines for other respective comparisons. Red bars represent rescue combinations. Lymph gland lobes are outlined with a white border and, for clarity, the background containing other tissues, such as ring gland, brain, dorsal vessel, etc., has been removed.

*Drosophila* lymph gland blood progenitor cells internalize systemic GABA (eGABA) via the GABA transporter (Gat) and catabolize it into succinate via the GABA catabolic pathway (Fig. 1A) (Madhwal et al., 2020). Succinic-semialdehyde dehydrogenase (Ssadh), the final and rate-limiting step of the GABA catabolic pathway (Shelp et al., 1999), is responsible for the generation of succinate in progenitor cells.

We observed that the loss of components of the GABA catabolic pathway (Fig. 1A) from blood progenitor cells led to a significant reduction in overall size of the lymph gland (Fig. 1B-D,H). Although defects in lymph gland growth and progenitor homeostasis were evident in our previous study (Madhwal et al., 2020), the mechanism underlying the growth defect remained unaddressed. In this study, using a RNAi-mediated genetic knockdown approach, we downregulated each respective component of the GABA catabolic pathway in blood progenitor cells. Using blood progenitor-specific drivers (*domeMeso>GFP* and *TepIV>mCherry*), we assessed its role in homeostatic conditions. For this, we blocked the following: (1) progenitor cell *Gat* function, in order to perturb GABA uptake (Fig. 1C,H and Fig. S1A); and (2) *Ssadh*, to perturb its breakdown into succinate (Fig. 1D,H and Fig. S1A,B). Under these conditions, a significant reduction in lymph gland size was noticed.

On the other hand, in differentiating blood cells that were positive for the blood maturation marker hemolectin (Hml^+^), a relatively lower level of Gat protein expression was seen, in comparison with the adjoining Hml^−^ progenitor cells that showed higher Gat expression (Fig. S1C,D). Furthermore, we also observed that blocking GABA uptake in Hml^+^ differentiating blood cells showed no changes in lymph gland growth [they remained comparable with control sizes (Fig. S1E-G)]. This demonstrated a specific function for GABA breakdown in progenitor cells in the control of lymph gland growth.

The final metabolic output of GABA breakdown is succinate (Fig. 1A). When larvae expressing *Gat^RNAi^* or *Ssadh^RNAi^* in blood progenitor cells were reared on food supplemented with succinate, they showed significant restoration of lymph gland sizes (Fig. 1E-H), which were almost comparable with sizes detected in control animals reared on a regular diet (Fig. 1F-H). Furthermore, controls raised on a succinate-supplemented diet also showed an ∼20% increase in lymph gland size in comparison with controls raised on a regular diet (Fig. 1E,H). This indicated a role for succinate, downstream of the GABA metabolic pathway, in moderating lymph gland size. This observation further led us to investigate the downstream mechanism by which this pathway controlled lymph gland growth.

### GABA catabolism in blood progenitor cells controls their ROS levels

**Fig. 2.**
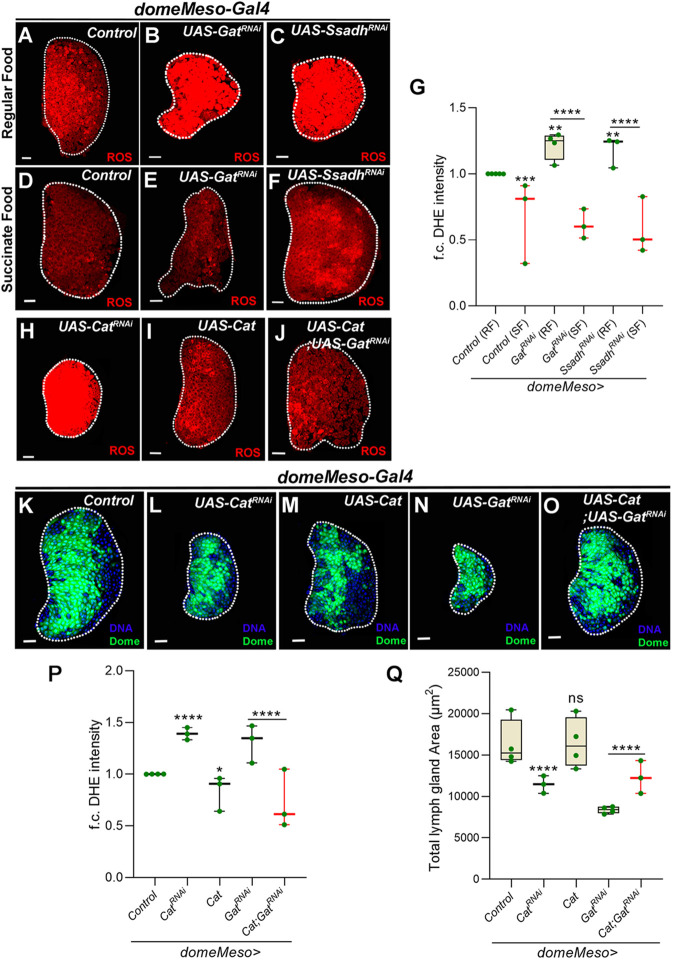
**ROS regulation by the GABA shunt pathway in *Drosophila* blood progenitors is important for lymph gland growth.** (A) Control (RF, *domeMeso-Gal4,UAS-GFP/+*) lymph gland showing higher ROS levels in the blood progenitor cells (dome^+^) when compared with the differentiating cells. (B) *Gat^RNAi^* (RF, *domeMeso-Gal4,UAS-GFP;UAS-Gat^RNAi^*) and (C) *Ssadh^RNAi^* (RF, *domeMeso-Gal4,UAS-GFP;UAS-Ssadh^RNAi^*) lead to increases in ROS levels when compared with A (control on RF). (D-F) Succinate supplementation of (D) control (*domeMeso-Gal4,UAS-GFP/+*), (E) *Gat^RNAi^* (*domeMeso-Gal4,UAS-GFP;UAS-Gat^RNAi^*) and (F) *Ssadh^RNAi^* (*domeMeso-Gal4,UAS-GFP;UAS-Ssadh^RNAi^*) leads to a reduction in ROS levels when compared with A-C, respectively. (G) Quantification of A-F. Relative fold change in lymph gland ROS (DHE) levels in *domeMeso>GFP/+* (control, RF, *N*=5, *n*=53), *domeMeso>GFP/+* (SF, *n*=27, *N*=3, *P*=0.0003), *domeMeso>GFP/Gat^RNAi^* (RF, *N*=4, *n*=30, *P*=0.0042), *domeMeso>GFP/Gat^RNAi^* (SF, *N*=3, *n*=22, *P*<0.0001), *domeMeso>GFP/Ssadh^RNAi^* (RF, *N*=3, *n*=28, *P*=0.0088) and *domeMeso>GFP/Ssadh^RNAi^* (SF, *N*=3, *n*=25, *P*<0.0001). (H-J) Expressing (H) *Catalase^RNAi^* (*domeMeso-Gal4,UAS-GFP;UAS-Cat^RNAi^*) leads to an increase in ROS levels; over-expressing (I) *Catalase* (*domeMeso-Gal4,UAS-GFP;UAS-Cat*) shows the reduction in ROS levels when compared with A (control); and overexpressing (J) *Catalase* in *Gat^RNAi^* (*domeMeso-Gal4,UAS-GFP;UAS-Cat;UAS-Gat^RNAi^*) rescues the lymph gland ROS defect of B (*Gat^RNAi^*). (K-O) Representative images showing lymph gland size. (K) Control (*domeMeso-Gal4,UAS-GFP*/+). (L) *Cat^RNAi^* in blood progenitors (*domeMeso-Gal4,UAS-GFP;UAS-Cat^RNAi^*) produces a reduction in lymph gland size. (M) Overexpressing *Catalase* (*domeMeso-Gal4,UAS-GFP;UAS-Cat*) produces no change in lymph gland size when compared with K (control). (N) *Gat*^*RNAi*^ produces a reduction in lymph gland size. (O) Overexpressing *Catalase* in *Gat^RNAi^* (*domeMeso-Gal4,UAS-GFP;UAS-Cat*;*UAS-Gat^RNAi^*) leads to rescue of the lymph gland size defect when compared with N (*Gat^RNAi^*). (P) Relative fold change in lymph gland ROS (DHE) levels in *domeMeso>GFP/+* (control, *N*=4, *n*=40), *domeMeso>GFP/Cat^RNAi^* (*N*=3, *n*=30, *P*<0.0001), *domeMeso>GFP/Cat* (*N*=3, *n*=28, *P*=0.0455), *domeMeso>GFP/Gat^RNAi^* (*N*=3, *n*=28, *P*=0.0003) and *domeMeso>GFP/Cat;Gat^RNAi^* (*N*=3, *n*=23, *P*<0.0001). (Q) Quantification of lymph gland area in K-O: *domeMeso>GFP/+* (control, *N*=4, *n*=40), *domeMeso>GFP/Cat^RNAi^* (*N*=3, *n*=45, *P*<0.0001), *domeMeso>GFP/Cat* (*N*=4, *n*=37, *P*>0.9999), *domeMeso>GFP/Gat^RNAi^* (*N*=4, *n*=35, *P*<0.0001) and *domeMeso>GFP/Cat;Gat^RNAi^* (*N*=3, *n*=37, *P*<0.0001). RF, regular food; SF, succinate-supplemented food. The horizontal line indicates the median; whiskers indicate the minimum and maximum values; box indicates the lower and upper quartiles (*P<0.05, **P<0.01, ***P<0.001, ****P<0.0001; n.s., not significant; two-way ANOVA, Tukey's multiple comparisons test). f.c., fold change. Scale bars: 20 µm. *n*, lymph gland lobes; *N*, number of experimental repeats (green dot). DAPI marks DNA. Comparisons for significance are with control values unless marked by horizontal lines for other respective comparisons; red bars represent rescue combinations. Lymph gland lobes are outlined with a white border and, for clarity, the background containing other tissues, such as ring gland, brain, dorsal vessel, etc., has been removed.

The progenitor cells of the lymph gland exhibit elevated levels of ROS (Fig. 2A), which are necessary for their maintenance and differentiation (Owusu-Ansah and Banerjee, 2009). We observed that blocking *Gat* or *Ssadh* function in blood progenitor cells led to elevation of lymph gland ROS (Fig. 2A-C,G). This implicated intracellular GABA uptake and its breakdown in blood progenitor ROS homeostasis. We tested the involvement of succinate as the metabolic output of GABA breakdown in progenitor ROS modulation. Similar to the growth phenotype, mutant lymph glands expressing *Gat^RNAi^* or *Ssadh^RNAi^* showed a significant downregulation of ROS levels when reared on a succinate-supplemented diet (Fig. 2D-G). Importantly, control animals raised on the succinate-supplemented diet showed a further reduction in progenitor ROS levels when compared with animals reared on regular food (Fig. 2D,G). Together, these data suggested that succinate derived from GABA metabolism was sufficient for moderating ROS in progenitor cells and suggested an underlying connection between GABA breakdown, succinate generation and ROS levels in the control of lymph gland growth.

To address whether elevated ROS detected in GABA metabolic mutants was indeed the reason for lymph gland growth retardation, we investigated whether increasing progenitor ROS through independent means impacted lymph gland size. For this, we performed RNAi against ROS scavenging enzymes, *Catalase* (*Cat*) and *Superoxide dismutase 2* (*Sod2*) in progenitor cells. Similar to *Gat* and *Ssadh* loss of function, knockdown of these scavenging enzymes raised levels of ROS (Fig. 2H,P and Fig. S2A,B,G) with a concomitant reduction in lymph gland size (Fig. 2K,L,Q and Fig. S2H). This suggested that conditions leading to elevated progenitor ROS negatively affected lymph gland growth. To substantiate the above finding, we performed experiments to investigate whether scavenging ROS by feeding *Gat* and *Ssadh* knockdown larvae with N-acetylcysteine (NAC), a known antioxidant (Niraula and Kim, 2019), was sufficient to recover their growth defect. As predicted, we observed restoration of ROS levels in the mutant conditions (Fig. S2C-G) with a significant recovery of lymph gland size (Fig. S2H,I). Furthermore, we also overexpressed a ROS scavenging enzyme, *Cat*, in progenitor cells with and without the co-expression of *Gat^RNAi^*. Although overexpression of *Cat* in progenitor cells led to downregulation of ROS (Fig. 2I,P), it did not alter lymph gland size (Fig. 2M,Q). However, co-expressing *UAS-Cat* in the *Gat^RNAi^* condition demonstrated a significant reduction in ROS (Fig. 2J,P) concurrent with a recovery in lymph gland size (Fig. 2N,O,Q and Fig. S2I). These data suggested that, in homeostasis, lowering ROS beyond the basal threshold did not lead to any growth advantage. However, in the GABA loss condition, the elevation in ROS above physiological levels led to the growth defect.

To show that the growth recovery seen in these genetic combinations was not a consequence of reducing Gal4 activity or Gal4 dilution, we assessed the level of GFP expression (*UAS-GFP*) in these lymph glands. *UAS-GFP* is co-expressed along with the different transgenes under the control of the same progenitor-specific *Gal4* (*domeMeso-Gal4*) and therefore forms a reliable read-out of Gal4 activity. We observed comparable GFP across all genetic combinations (Fig. S2J), which suggested uniform *Gal4* activity. This further confirmed our conclusions, showed that the maintenance of ROS homeostasis was mediated by GABA metabolism in progenitor cells and that this was crucial for lymph gland growth.

### TCA activity: a prime producer of ROS in blood progenitor cells

**Fig. 3.**
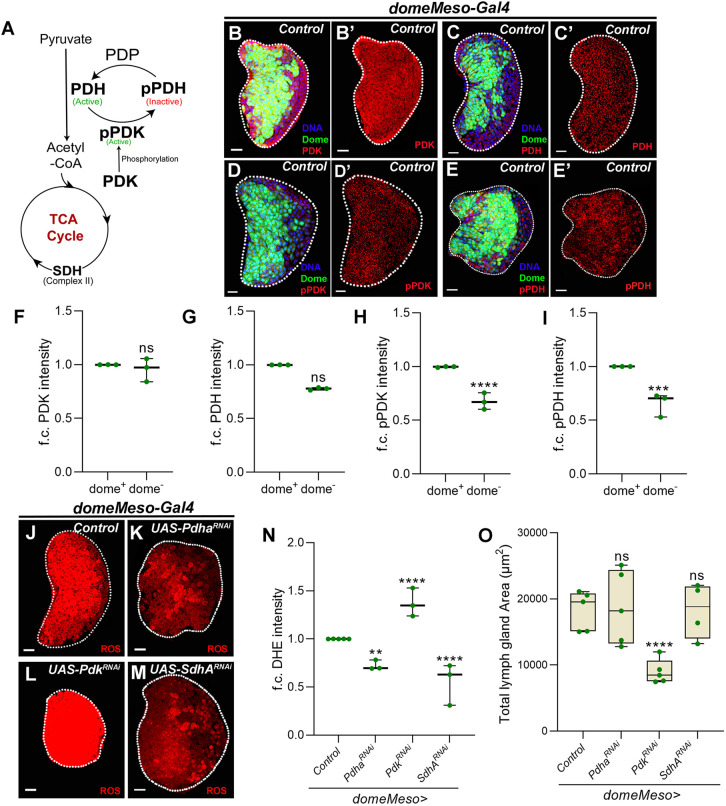
**TCA cycle activity contributes to blood progenitor ROS levels and regulates lymph gland growth.** (A) Schematic representation showing regulation of pyruvate entry and conversion to acetyl-CoA by PDH enzyme (active). PDH enzyme is phosphorylated by pPDK, which makes it inactive; PDP dephosphorylates pPDH (inactive) to PDH (active), which converts pyruvate into acetyl-CoA and fuels the TCA cycle. (B-C′) Representative lymph gland images showing PDK (red) and PDH (red) in control lymph glands (*domeMeso-Gal4,UAS-GFP/+*). (B,B′) PDK expression and (C,C′) PDH expression in the dome^+^ overlap (green, progenitors) and without dome^+^ overlap (non-green, differentiating cells) are uniform. (D-E′) Representative lymph gland images showing pPDK (red) and pPDH (red) in control lymph glands (*domeMeso-Gal4,UAS-GFP/+*). (D,D′) pPDK and (E,E′) pPDH in the dome^+^ overlap (green, progenitors) and without dome^+^ overlap (non-green, differentiating cells), respectively. dome^+^ cells show more pPDK and pPDH when compared with the dome^−^ cells. (F-I) Quantification of A-E′. Relative fold change in (F) PDK levels in *domeMeso>GFP/+* in the dome^+^ (*N*=3, *n*=15) and dome^−^ region (*N*=3, *n*=15, *P*=0.7622), (G) PDH levels in *domeMeso>GFP/+* in the dome^+^ (*N*=3, *n*=15) and dome^−^ region (*N*=3, *n*=15, *P*=0.0595), (H) pPDK levels in *domeMeso>GFP/+* in the dome^+^ (*N*=3, *n*=17) and dome^−^ region (*N*=3, *n*=17, *P*<0.0001), and (I) pPDH levels in *domeMeso>GFP/+* in the dome^+^ (*N*=3, *n*=18) and dome^−^ region (*N*=3, *n*=18, *P*=0.0001). (J-M) Representative lymph gland images showing ROS levels. (J) Control (*domeMeso-Gal4,UAS-GFP*/+). (K) *Pdha^RNAi^* (*domeMeso-Gal4,UAS-GFP;UAS-Pdha^RNAi^*) leads to a reduction in ROS levels, (L) *Pdk^RNAi^* (*domeMeso-Gal4,UAS-GFP;UAS-Pdk^RNAi^*) elevates ROS levels and (M) *SdhA^RNAi^* (*domeMeso-Gal4,UAS-GFP;UAS-SdhA^RNAi^*) leads to a reduction in ROS levels when compared with J (control). (N) Quantification of J-M. Relative fold change in lymph gland ROS (DHE) levels in *domeMeso>GFP*/+ (control, *N*=5, *n*=50), *domeMeso>GFP*/*Pdha^RNAi^* (*N*=3, *n*=32, *P*=0.0019), *domeMeso>GFP*/*Pdk^RNAi^* (*N*=3, *n*=36, *P*<0.0001) and *domeMeso>GFP*/*SdhA^RNAi^* (*N*=3, *n*=24, *P*<0.0001). (O) Quantifications of lymph gland size in *domeMeso>GFP*/+ (control, *N*=5, *n*=44), *domeMeso>GFP*/*Pdha^RNAi^* (*N*=5, *n*=54, *P*>0.9999), *domeMeso>GFP*/*Pdk^RNAi^* (*N*=5, *n*=51, *P*<0.0001) and *domeMeso>GFP*/*SdhA^RNAi^* (*N*=4, *n*=42, *P*=0.9887). The horizontal line indicates the median; whiskers indicate the xxxxx; box indicates the xxxxx (**P<0.01, ***P<0.001, ****P<0.0001; n.s., not significant; two-way ANOVA, Tukey's multiple comparisons test). f.c., fold change. Scale bars: 20 µm. *n*, lymph gland lobes; *N*, number of experimental repeats (green dot). DAPI marks DNA. Lymph gland lobes are outlined with a white border and, for clarity, the background containing other tissues, such as ring gland, brain, dorsal vessel, etc., has been removed.

Next, we investigated the mechanism underlying progenitor ROS homeostasis via GABA. To explore this, we first examined the source of developmental ROS in these cells. The TCA cycle and its intermediates, which drive mitochondrial oxidative phosphorylation (OXPHOS), are a significant center for ROS production (Quinlan et al., 2012; Woolbright et al., 2019). Therefore, we investigated TCA cycle activity in lymph gland progenitor cells. As a proxy for measuring TCA cycle activity, we assessed levels of the enzymes PDK and PDH using antibodies that detected their total levels along with active and inactive forms (Fig. 3), respectively. PDH enzyme converts pyruvate to acetyl-CoA and drives the TCA cycle (Wang et al., 2016). PDH activity is regulated at the level of its phosphorylation, where the phosphorylated form (pPDH) is an inactive enzyme that represses TCA cycle activity. Phosphorylation of PDH is mediated by PDK. The phosphorylated form of PDK (pPDK) is an active form that inhibits PDH, thereby decreasing TCA cycle activity (Fig. 3A).

Immunohistochemical analysis of 3rd instar larval lymph glands against PDK^total^, PDH^total^, active pPDK and inactive pPDH was performed. PDK^total^ (Fig. 3B,B′,F) and PDH^total^ (Fig. 3C,C′,G) showed uniform expression in all cells of a 3rd instar larval lymph gland. However, the levels of pPDK and pPDH were specifically elevated in dome^+^ progenitor cells in comparison with the levels detected in the dome^−^ differentiating cells (Fig. 3D-E′,H,I). These data suggested that, in homeostasis, blood progenitor cells maintained a substantial fraction of PDH in an inactive state (pPDH). The increased threshold of active PDK (pPDK) in progenitor cells also suggested that PDK was regulated to limit PDH function and played a role in the moderation of TCA cycle activity.

We employed genetic methods to perturb PDH and PDK enzymes by performing RNAi, followed by assessment for changes in progenitor ROS generation and consequently blood progenitor development. First, we confirmed the specificity of the RNAi lines by undertaking an analysis of PDH^total^ and pPDH in lymph glands expressing *Pdha*^*RNAi*^ (pyruvate dehydrogenase E1 alpha subunit) and *Pdk^RNAi^* (Fig. S3). A striking downregulation of PDH^total^ (Fig. S3A,B,G) and pPDH (Fig. S3D,E,H) was seen in *Pdha^RNAi^*-expressing lymph glands. In *Pdk^RNAi^*-expressing lymph glands, PDH^total^ remained unaffected (Fig. S3C,G), but the levels of the phosphorylated form (pPDH) were reduced (Fig. S3F,H). Together, these data confirmed the specificity of the RNAi lines. The specific loss of pPDH in the *Pdk^RNAi^* condition also showed that the phosphorylation of PDH in progenitor cells was reliant on PDK function.

RNAi-mediated knockdown of *Pdha* expression in blood progenitor cells, led to an almost 50% reduction in ROS compared with levels detected in control lymph glands (Fig. 3J,K,N). Importantly, loss of *Pdha* expression in the progenitors did not impede lymph gland growth and they were comparable with the control conditions (Fig. 3O). These data were consistent with *Cat* overexpression results (Fig. 2K,Q), which further strengthened the notion that lowering progenitor ROS below physiological levels did not lead to any growth advantage. On the other hand, blocking *Pdk* expression in blood progenitor cells, using two independent RNAi lines, led to an ∼1.5-fold increase in ROS (Fig. 3L,N and Fig. S3I) and a lymph gland growth defect (Fig. 3O and Fig. S3J). These data were consistent with loss of *Gat*, *Ssadh*, *Cat* or *Sod2* conditions, where an increase in ROS accompanied the reduction in lymph gland size (Fig. 2K,Q and Fig. S2G,H). Taken together, *Pdha* loss-of-function data suggested that PDH function facilitated the production of ROS in progenitor cells, while *Pdk* loss-of-function data showed that the role of PDK activity in modulating progenitor ROS levels was necessary for normal lymph gland growth.

Consistent with the involvement of the TCA cycle in OXPHOS, progenitor-specific downregulation of the TCA cycle enzyme gene *Succinate dehydrogenase A* (*SdhA*), which is also a component of Complex II of the mitochondrial electron transport chain (ETC), lowered progenitor ROS levels (Fig. 3M,N). In this genetic condition, lymph gland sizes also remained unaffected (Fig. 3O) and the data were consistent with phenotypes detected with *Pdha* that functioned upstream of the TCA cycle. The data showed that, in homeostatic conditions, PDH-dependent entry of pyruvate into TCA and the subsequent activation of mitochondrial ETC via SDH caused ROS production in blood progenitor cells.

Next, we examined whether modulation of TCA enzymes affected progenitor homeostasis and immune response. The effect on progenitor homeostasis was addressed by analyzing the *dome-GFP* reporter in the progenitor cells and by staining lymph glands for differentiation markers P1, to mark plasmatocytes (Fig. S4A-E), and PPO1, to mark crystal cells (Fig. S4F) (Krzemien et al., 2010). The immune response was assessed by analyzing the formation of lamellocytes using a myospheroid marker in response to parasitic wasp infections (Irving et al., 2005). Lamellocyte numbers were assessed in the lymph glands and in circulation at 24 h (Fig. S4G) and 48 h (Fig. S4H) post-wasp infection, respectively. In control lymph glands, a 60-70% area of lymph gland was dome^+^, a 15-20% area was dome^−^ but P1^+^ and the remaining 20-25% area was negative for both the markers (dome^−^P1^−^) (Fig. S4A-A″,E). Loss of *Pdha* (low TCA) from progenitor cells did not alter progenitor homeostasis or differentiation, which remained comparable with controls (Fig. S4A-B″,E,F). Like *Pdha* loss of function, *SdhA^RNAi^* did not produce any dramatic changes in progenitor maintenance or their differentiation status, except a mild reduction in progenitor population (Fig. S4C-C″,E,F). When assessed for immune response post-wasp infection, the loss of TCA cycle function did not impede lamellocyte formation. In contrast, a significant increase in lamellocyte numbers was detected in the lymph glands lacking *Pdha* or *SdhA* expression (Fig. S4G,H). These data revealed an unexpected role for TCA cycle regulation in the immune response, while being largely dispensable for normal hematopoiesis. In contrast, in the *Pdk^RNAi^* condition (increased TCA), the changes in the progenitor population were more dramatic. We observed an increase in the dome^+^ population along with an increase in the P1^+^ population and a reduction in dome^−^P1^−^ population (Fig. S4D-E). Moreover, a subset of dome^+^ cells that overlapped with P1^+^ cells (Fig. S4D-E) was observed, which was otherwise undetectable in control lymph glands under homeostasis. A reduction in crystal cell formation was also evident in the *Pdk^RNAi^* condition (Fig. S4F). When assessed for cellular immune response, loss of *Pdk* function severely abrogated lamellocyte formation (Fig. S4G,H). These data also suggested a role for PDK in progenitor maintenance and differentiation in homeostasis, and for immune response. The *Pdk^RNAi^* immune phenotypes were comparable with phenotypes described for GABA function in blood progenitor cells (Madhwal et al., 2020). Although, in homeostasis, loss of GABA metabolism has not been shown to dramatically impede progenitor maintenance (Madhwal et al., 2020), a careful assessment of *Gat^RNAi^* lymph glands with the markers described in this study revealed an overlap between dome^+^ cells and P1^+^ marker (Figs S4E and S5D-D″,G). This suggested a functional overlap between GABA catabolism and PDK activity in regulating lymph gland growth, progenitor differentiation and the immune response.

### GABA catabolism regulates TCA activity by regulating PDK function and moderates ROS generation in blood progenitor cells

**Fig. 4.**
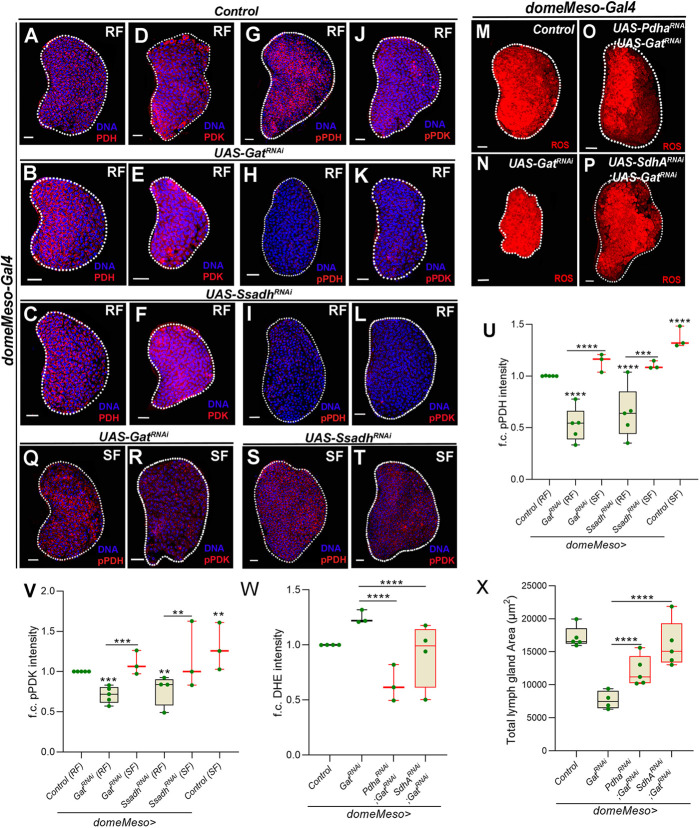
**GABA catabolism via PDK activity regulates the TCA cycle in blood progenitor cells and coordinates overall lymph gland growth.** (A-F) Representative lymph gland images showing PDH and PDK levels. (A,D) Controls (*domeMeso-Gal4,UAS-GFP/+*) showing (A) PDH and (D) PDK levels. (B,E) *Gat^RNAi^* (*domeMeso-Gal4,UAS-GFP;UAS-Gat^RNAi^*) and (C,F) *Ssadh^RNAi^* (*domeMeso-Gal4,UAS-GFP;UAS-Ssadh^RNAi^*) in progenitor cells does not reduce (B,C) PDH and (E,F) PDK levels when compared with A and D, respectively. For quantification, refer to Fig. S5A,B. (G-I) Representative lymph gland images showing pPDH on RF. (G) Control (*domeMeso-Gal4,UAS-GFP/+*). (H) *Gat^RNAi^* (*domeMeso-Gal4,UAS-GFP;UAS-Gat^RNAi^*) and (I) *Ssadh^RNAi^* (*domeMeso-Gal4,UAS-GFP;UAS-Ssadh^RNAi^*) lead to a reduction in pPDH levels when compared with G (control). (J-L) Representative lymph gland images showing pPDK on RF. (J) Control (*domeMeso-Gal4,UAS-GFP/+*). (K) *Gat^RNAi^* (*domeMeso-Gal4,UAS-GFP;UAS-Gat^RNAi^*) and (L) *Ssadh^RNAi^* (*domeMeso-Gal4,UAS-GFP;UAS-Ssadh^RNAi^*) lead to a reduction in pPDK levels when compared with J (control). (M-P) Representative lymph gland images showing ROS levels. (M) Control (*domeMeso-Gal4,UAS-GFP/*+). (N) *Gat^RNAi^* (*domeMeso-Gal4,UAS-GFP;UAS-Gat^RNAi^*) leads to an elevation in ROS levels. (O) *Pdha^RNAi^;Gat^RNAi^* (*domeMeso-Gal4,UAS-GFP;UAS-Pdha^RNAi^;UAS-Gat^RNAi^*) and (P) *SdhA^RNAi^;Gat^RNAi^* (*domeMeso-Gal4,UAS-GFP;UAS-SdhA^RNAi^;UAS-Gat^RNAi^*) rescue the increased ROS phenotype of N (*Gat^RNAi^*). (Q-T) Succinate supplementation (SF) in (Q,R) *Gat^RNAi^* (*domeMeso-Gal4,UAS-GFP;UAS-Gat^RNAi^*) and (S,T) *Ssadh^RNAi^* (*domeMeso-Gal4,UAS-GFP;UAS-Ssadh^RNAi^*) rescues (Q,S) pPDH and (R,T) pPDK levels of (H,K) *Gat^RNAi^* and (I,L) *Ssadh^RNAi^* on RF. (U) Quantification of G-I,Q,S. Relative fold change in lymph gland MZ pPDH levels in *domeMeso>GFP*/+ (RF, control, *N*=5, *n*=51), *domeMeso>GFP/Gat^RNAi^* (RF, *N*=5, *n*=32, *P*<0.0001), *domeMeso>GFP/Gat^RNAi^* (SF, *N*=3, *n*=24, *P*<0.0001), *domeMeso>GFP/Ssadh^RNAi^* (RF, *N*=5, *n*=47, *P*<0.0001), *domeMeso>GFP/Ssadh^RNAi^* (SF, *N*=3, *n*=23, *P*=0.0007) and *w^1118^* (SF, *N*=3, *n*=38, *P*<0.0001, pPDH compared with *w^1118^*, *N*=3, RF, *n*=47). (V) Quantification of J-L,R,T. Relative fold change in lymph gland MZ pPDK levels in *domeMeso>GFP*/+ (RF, control, *N*=5, *n*=75), *domeMeso>GFP*/*Gat^RNAi^* (RF, *N*=5, *n*=38, *P*=0.0004), *domeMeso>GFP*/*Gat^RNAi^* (SF, *N*=3, *n*=20, *P*=0.0006), *domeMeso>GFP*/*Ssadh^RNAi^* (RF, *N*=4, *n*=35, *P*=0.0058), *domeMeso>GFP*/*Ssadh^RNAi^* (SF, *N*=3, *n*=20, *P*=0.0021) and *w^1118^* (SF, *n*=30, *P*=0.0071, pPDK compared with *w^1118^*, RF, *N*=3, *n*=30). (W) Quantification of M-P. Relative fold change in lymph gland ROS (DHE) levels in *domeMeso>GFP/+*(control, *N*=4, *n*=38), *domeMeso>GFP/Gat^RNAi^* (*N*=3, *n*=22, *P*<0.0001), *domeMeso>GFP/Pdha^RNAi^;Gat^RNAi^* (*N*=3, *n*=19, *P*<0.0001) and *domeMeso>GFP/SdhA^RNAi^;Gat^RNAi^* (*N*=4, *n*=26, *P*<0.0001). (X) Quantifications of lymph gland size in *domeMeso>GFP/+* (control, *N*=5, *n*=44), *domeMeso>GFP/Gat^RNAi^* (*N*=4, *n*=36, *P*<0.0001), *domeMeso>GFP/Pdha^RNAi^;Gat^RNAi^* (*N*=5, *n*=50, *P*<0.0001), and *domeMeso>GFP/SdhA^RNAi^;Gat^RNAi^* (*N*=5, *n*=41, *P*<0.0001). RF, regular food; SF is succinate-supplemented food. The horizontal line indicates the median; whiskers indicate the minimum and maximum values; box indicates the lower and upper quartiles (**P<0.01, ***P<0.001, ****P<0.0001; n.s., not significant; two-way ANOVA, Tukey's multiple comparisons test). f.c., fold change. Scale bars: 20 µm. *n*, lymph gland lobes; *N*, number of experimental repeats (green dot). MZ, medullary zone. DAPI marks DNA. Comparisons for significance are with control values unless marked by horizontal lines for other respective comparisons; red bars represent rescue combinations. Lymph gland lobes are outlined with a white border and, for clarity, the background containing other tissues, such as ring gland, brain, dorsal vessel, etc., has been removed.

Based on the phenotypic similarities between GABA and *Pdk* loss of function, we hypothesized that an increase in TCA cycle activity in GABA catabolic mutants led to the small lymph gland and differentiation phenotypes. To test this, we investigated the levels of PDH^total^, PDK^total^, the inactive form of PDH (pPDH) and active PDK (pPDK) in GABA metabolic pathway mutants (Fig. 4 and Fig. S5). PDH^total^ and PDK^total^ expression in the progenitor cells upon *Gat* and *Ssadh* knockdown remained comparable with controls (Fig. 4A-F and Fig. S5A,B). This showed that changes in GABA metabolism did not alter the production of these enzymes. However, we observed a specific downregulation of the levels of pPDH (Fig. 4G-I,U) and pPDK (Fig. 4J-L,V) upon loss of *Gat* and *Ssadh* expression in progenitor cells. The specific reduction of pPDK in GABA metabolic mutants implied that the GABA metabolic pathway functioned in maintaining an active PDK state in progenitor cells, as a means to limit PDH activity and consequently suppress the TCA cycle. This notion was supported by the reduction in pPDH levels also seen in the mutant lymph glands, which suggested an increased fraction of active PDH enzyme that most likely led to enhanced TCA cycle activity in these mutants. We therefore investigated whether downregulating components of the TCA cycle in the *Gat^RNAi^* condition could restore lymph gland ROS and growth phenotypes. Interestingly, downregulation of *Pdha* or *SdhA* expression in *Gat^RNAi^* progenitor cells corrected the ROS phenotype to levels detected in control (Fig. 4M-P,W) and also restored lymph gland size (Fig. 4X). This implied that the increased TCA cycle activity in GABA metabolic mutants was the source of aberrant ROS generation that led to the growth defect. The downregulation of *Pdha* or *SdhA* in the *Gat^RNAi^* condition also recovered the blood progenitor differentiation and immune defects seen in *Gat^RNAi^* animals (Fig. S5C-J). This included the restoration of dome^+^P1^+^ double-positive cells detected in the *Gat^RNAi^* condition that were no longer detectable in *Pdha^RNAi^;Gat^RNAi^* or *SdhA^RNAi^;Gat^RNAi^* conditions (Fig. S5C-G). The lamellocyte formation defect seen in *Gat^RNAi^* (Fig. S5I,J) was also significantly restored upon knockdown of *Pdha* or *SdhA* (Fig. S5I,J). These data suggested that GABA function in progenitor cells via regulating PDK activity controlled PDH-dependent pyruvate entry into the TCA cycle and OXPHOS, and this limited the generation of excessive ROS. This regulation supported the homeostatic growth and differentiation of lymph gland blood progenitor cells. Given the striking recovery of the lamellocyte response with the loss of TCA cycle components in the *Gat^RNAi^* condition, we also tested whether correcting ROS could restore their immune response. This was undertaken by driving *Cat* overexpression in *Gat^RNAi^* condition, but this combination failed to show any recovery in lamellocyte number (Fig. S5I,J). These data showed that, while excessive TCA activity in the *Gat^RNAi^* condition blocked lamellocyte formation, this inhibition was not due to the elevated ROS seen in them. The data suggested a role for GABA metabolism in the control of TCA activity in mounting a successful immune response, which was independent of ROS generation.

### GABA catabolism via succinate controls the PDK activity necessary for ROS homeostasis in blood progenitor cells

Next, we investigated the mechanism by which the GABA catabolic pathway regulates pPDK levels in the progenitors. The restoration of ROS and the lymph gland growth defect by a succinate-supplemented diet implied regulation of the TCA cycle by succinate (Figs 1 and 2A-G). We examined the levels of pPDH and pPDK in succinate-supplemented conditions. In comparison with pPDH and pPDK levels detected in *Gat* and *Ssadh* RNAi animals raised on regular food (Fig. 4G-L,U,V), the RNAi animals fed on a succinate-supplemented diet showed restored levels of pPDH (compare Fig. 4Q,S with H,I, respectively; Fig. 4U) and pPDK (compare Fig. 4R,T with K,L, respectively; Fig. 4V) that were almost comparable with levels seen in control lymph glands. This implied that GABA-derived succinate in progenitors activated PDK function to moderate TCA cycle activity and lower ROS generation. This was an unexpected finding, as cycling of succinate in the TCA cycle, via its conversion through *Sdha* in progenitor cells, was necessary to promote ROS generation (Fig. 3M,N). These data therefore suggested that GABA metabolism-derived succinate was independent of the TCA cycle-derived succinate, and that these two pools functioned distinctly to coordinate lymph gland ROS levels. While the TCA cycle-derived succinate drove progenitor cell ROS generation, GABA-derived succinate opposed it.

The mechanism by which GABA catabolism to succinate regulated ROS was examined next. Our previous findings implicate GABA catabolism-derived succinate in the inhibition of hydroxy prolyl hydroxylase (Hph), which is necessary for cellular immune response to wasp infection. We have shown that, in conditions of low GABA metabolism in progenitor cells, the reduction in succinate causes an increase in Hph function and abrogation of the cellular immune response. In contrast to this, increased GABA metabolism caused reduction of Hph and led to superior immune responses (Madhwal et al., 2020). Therefore, in the context of lymph gland growth, we investigated whether GABA and succinate also function by moderating Hph.

**Fig. 5.**
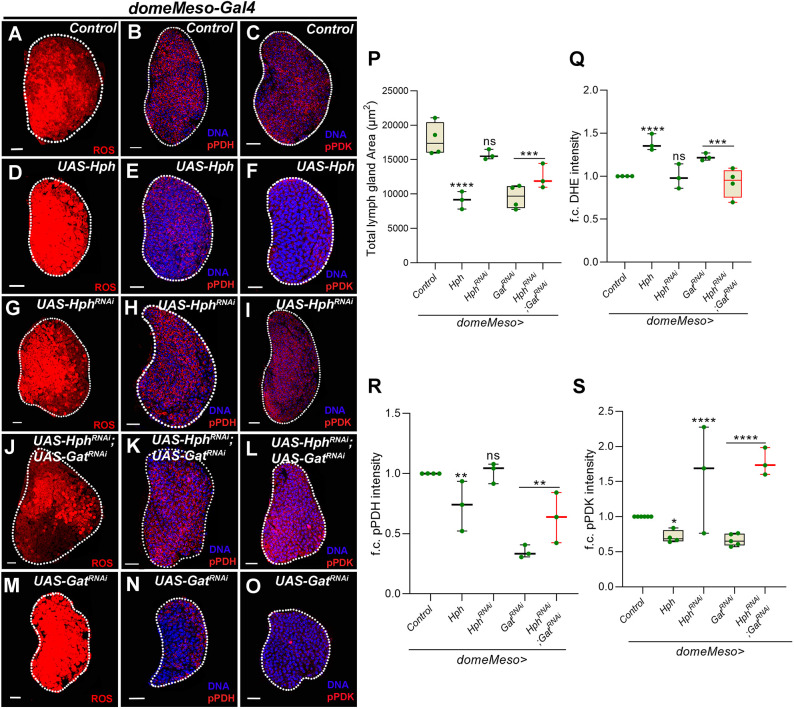
**GABA catabolism-derived succinate inhibits Hph function to maintain PDK activity and limit the TCA cycle, which sustains lymph gland growth.** (A-C) Representative lymph gland images showing (A) ROS (B) pPDH and (C) pPDK in control (*domeMeso-Gal4,UAS-GFP/+*). (D-F) Overexpressing *Hph* (*domeMeso-Gal4,UAS-GFP;UAS-Hph*) leads to (D) a significant increase in ROS levels, and a reduction in (E) pPDH and (F) pPDK levels in the progenitor cells. (G-I) Expressing *Hph^RNAi^* (*domeMeso-Gal4,UAS-GFP;UAS-Hph^RNAi^*) did not show any change in (G) ROS levels or (H) pPDH levels, but did show a significant increase in (I) pPDK levels in the progenitor cells. (J-O) Expressing *Hph^RNAi^* in *Gat^RNAi^* (*domeMeso-Gal4,UAS-GFP;UAS-Hph^RNAi^*;*UAS-Gat^RNAi^*) leads to (J) a reduction in lymph gland ROS levels when compared to *Gat^RNAi^* (M), and to an increase in (K) pPDH and (L) pPDK levels in the progenitor cells when compared with N and O (*domeMeso-Gal4,UAS-GFP;UAS-Gat^RNAi^*), respectively. (P) Quantification of lymph gland area in *domeMeso>GFP/+* (control, *N*=4, *n*=40), *domeMeso>GFP/Hph* (*N*=3, *n*=40, *P*<0.0001), *domeMeso>GFP/Hph^RNAi^* (*N*=3, *n*= 40, *P*=0.2262), *domeMeso>GFP/Gat^RNAi^* (*N*=4, *n*=40, *P*<0.0001) and *domeMeso>GFP/Hph^RNAi^;Gat^RNAi^* (*N*=3, *n*=40, *P*=0.0002). (Q) Quantification of A,D,G,J,M. Relative fold change in lymph gland ROS (DHE) levels in *domeMeso>GFP/+* (control, *N*=4, *n*=36), *domeMeso>GFP/Hph* (*N*=3, *n*=32 *P*<0.0001), *domeMeso>GFP/Hph^RNAi^* (*N*=3, *n*=24, *P*>0.9999), *domeMeso>GFP/Gat^RNAi^* (*N*=3, *n*=14, *P*=0.0623) and *domeMeso>GFP/Hph^RNAi^;Gat^RNAi^* (*N*=4, *n*=31, *P*=0.0004). (R) Quantification of B,E,H,K,N. MZ pPDH levels in *domeMeso>GFP/+* (control, *N*=4, *n*=57), *domeMeso>GFP/Hph* (*N*=3, *n*=39, *P*=0.0045), *domeMeso>GFP/Hph^RNAi^* (*N*=3, *n*=37, *P*=0.9872), *domeMeso>GFP/Gat^RNAi^* (*N*=3, *n*=20, *P*<0.0001) and *domeMeso>GFP/Hph^RNAi^;Gat^RNAi^* (*N*=3, *n*=29, *P*=0.0082). (S) Quantification of C,F,I,L,O. MZ pPDK levels in *domeMeso>GFP/+* (control, *N*=6, *n*=76), *domeMeso>GFP/Hph* (*N*=4, *n*=37, *P*=0.0378), *domeMeso>GFP/Hph^RNAi^* (*N*=3, *n*=41, *P*<0.0001), *domeMeso>GFP/Gat^RNAi^* (*N*=5, *n*=29, *P*=0.0266) and *domeMeso>GFP/Hph^RNAi^;Gat^RNAi^* (*N*=3, *n*=36, *P*<0.0001). The horizontal line indicates the median; whiskers indicate the minimum and maximum values; box indicates the lower and upper quartiles (*P<0.05, **P<0.01, ***P<0.001, ****P<0.0001; n.s., not significant; two-way ANOVA, Tukey's multiple comparisons test). f.c., fold change. Scale bars: 20 µm. *n*, lymph gland lobes; *N*, number of experimental repeats (green dot). MZ, medullary zone. DAPI marks DNA. Comparisons for significance are with control values unless marked by horizontal lines for other respective comparisons; red bars represent rescue combinations. Lymph gland lobes are outlined with a white border and, for clarity, the background containing other tissues, such as ring gland, brain, dorsal vessel, etc., has been removed.

To achieve this, we used gain and loss-of-function approaches to moderate Hph expression in progenitor cells. We observed that increased *Hph* levels (*domeMeso>Hph*) led to a reduction in lymph gland size (Fig. 5P), an elevation in ROS (Fig. 5A,D,Q), and a reduction in pPDH (Fig. 5B,E,R) and pPDK levels (Fig. 5C,F,S). Total levels of PDH or PDK remained unaffected (Fig. S6A,B). This showed that *Hph* overexpression caused a reduction in the levels of pPDK (Fig. 5F,S) and subsequently pPDH (Fig. 5E,R) without altering the total levels of these proteins. Together, the data suggested that elevated *Hph* expression was sufficient to increase TCA rate, to lead to heightened ROS generation and to cause a lymph gland growth defect. Reduction in progenitor cell *Hph* expression, however, had no effect on lymph gland size (Fig. 5P), ROS levels (Fig. 5G,Q) or PDH activity, as pPDH levels remained unchanged (Fig. 5H,R) even though an increase in pPDK levels (Fig. 5I,S) was apparent. We assessed for total PDH and PDK, which also remain unchanged (Fig. S6A,B). Surprisingly, these data are in contrast to those from gain of Hph function and suggest that, in homeostasis, *Hph* function in progenitor cells is not necessary to moderate TCA cycle activity or lymph gland growth.

Given the phenotypic similarities between *Hph* gain of function and GABA catabolic mutants, we hypothesized that, in the absence of GABA, the growth defect could be due to increased Hph function. To test this, we co-expressed *Hph^RNAi^* in progenitor cells in the background of *Gat^RNAi^*. This led to a significant recovery of lymph gland size (Fig. 5P), ROS (compare Fig. 5J with M,Q), pPDH (compare Fig. 5K with N,R) and pPDK levels (compare Fig. 5L with O,S). These data demonstrated that, in the absence of GABA metabolism, the gain in *Hph* function above the basal threshold led to the downregulation of pPDK levels. This increased TCA cycle activity led to elevated ROS generation, which consequently affected lymph gland growth. However, whether GABA also inhibited *Hph* in homeostasis to maintain the TCA cycle and redox balance remains unclear. Interestingly, our data show a complex role for Hph, which could be driven by different isoforms (Acevedo et al., 2010), and our current findings with the approaches used (either the RNA*i* or *UAS-Hph*) are limited in this aspect. The involvement of the specific isoform/s of Hph in blood development and their regulation needs to be addressed.

Thus far, the data highlight the requirement for GABA metabolism in homeostatic control of lymph gland growth via ROS regulation. The consequence of increasing GABA levels in blood progenitor cells on lymph gland growth was also assessed. This was undertaken by overexpression of *Gat* in progenitor cells, which would enable them to internalize more GABA and raise its intracellular levels. This condition led to a significant increase in lymph gland size when compared with control tissue (Fig. S6C). In addition to this, *Gat* overexpression also led to a significant reduction in progenitor ROS levels compared with those seen in homeostasis (Fig. S6D-F). Consistent with this, a reduction in TCA cycle activity was also detected (Fig. S6G-N). These data suggested that upregulation of GABA uptake by blood progenitor cells produced an increase in lymph gland growth. While the regulation of TCA cycle activity and ROS levels in increased GABA conditions could be mediated by further downregulation of *Hph*, the mechanisms that led to the growth advantage remain unclear and need to be deciphered. Overall, the data implied a necessary and a sufficient role for GABA metabolism in lymph gland size regulation.

### Physiological regulation of GABA in lymph gland growth

**Fig. 6.**
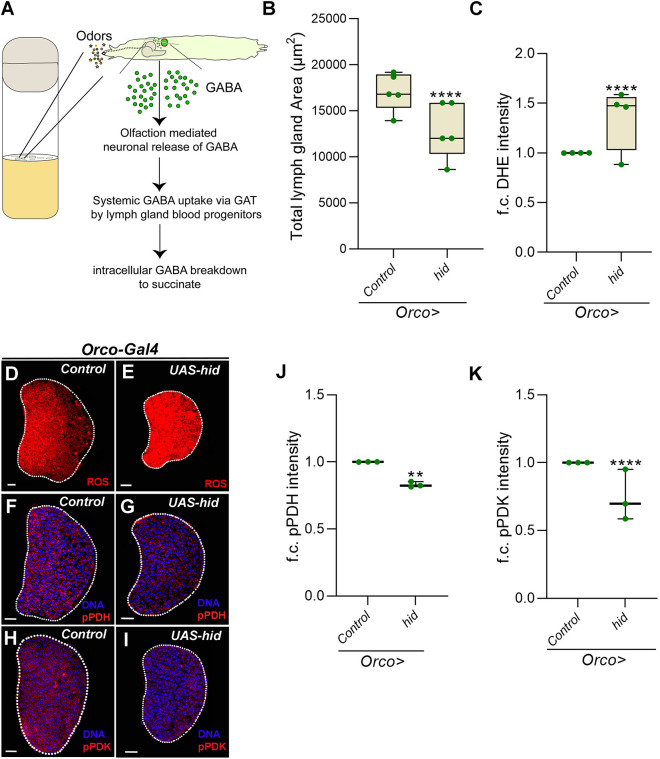
**The olfactory regulation of lymph gland growth and ROS homeostasis.** (A) A schematic representation showing larvae rearing in a food medium (shown in the vial) where the exposure of the animal to environmental odors stimulates the release of GABA from the larval brain. This extracellular GABA is sensed by blood progenitor cells of the lymph gland and is thereafter catabolized into succinate via the GABA catabolic pathway. (B) Quantifications of lymph gland size in *Orco>/+* (control, *N*=5, *n*=74) and *Orco>/hid* (*N*=5, *n*=76, *P*<0.0001). (C) Quantification of D,E. Relative fold change in lymph gland ROS (DHE) levels in *Orco>/+* (control, *N*=4, *n*=32) and *Orco>/hid* (*N*=4, *n*=30, *P*<0.0001). (D-I) Representative lymph gland images showing (D,E) ROS, (F,G) pPDH and (H,I) pPDK in controls (D,F,H; *Orco-Gal4/+*) and in *hid*-overexpressing animals (E,G,I). Overexpressing *hid* in olfactory receptor neurons (*Orco-Gal4;UAS-hid*) leads to (E) an increase in ROS levels, and a reduction in (G) pPDH and (I) pPDK levels when compared with D,F,H (controls), respectively. (J) Quantification of F,G. Relative fold change in lymph gland pPDH levels in *Orco>/+* (control, *N*=3, *n*=35) and *Orco>/hid* (*N*=3, *n*=29, *P*=0.0053). (K) Quantification of H,I. Relative fold change in lymph gland pPDK levels in *Orco>/+* (control, *N*=3, *n*=30) and *Orco>/hid* (*N*=3, *n*=33, *P*<0.0001). The horizontal line indicates the median; whiskers indicate the minimum and maximum values; box indicates the lower and upper quartiles (**P<0.01, ****P<0.0001; two-way ANOVA). f.c., fold change. Scale bars: 20 µm. *n*, lymph gland lobes; *N*, number of experimental repeats (green dot). DAPI marks DNA. Lymph gland lobes are outlined with a white border and, for clarity, the background containing other tissues, such as ring gland, brain, dorsal vessel, etc., has been removed.

The GABA detected by the lymph gland progenitor cells is derived from olfactory stimulation (Madhwal et al., 2020; Shim et al., 2013, Fig. 6A). As animals sense environmental odors, the olfactory input derived upon activation of specific olfactory receptors stimulates neuronal production and release of GABA, which is sensed by blood progenitor cells both as a signaling ligand (Shim et al., 2013) and as a metabolite (Madhwal et al., 2020). Our recent findings have implicated the metabolic role of GABA in the specification of immune cells necessary to respond to wasp infections (Madhwal et al., 2020). Whether the odor-sensing/GABA axis in physiological conditions moderated hematopoietic growth was therefore examined. For this, we assessed the impact of anosmia on lymph gland growth, the TCA cycle and ROS homeostasis. We employed genetic means to ablate olfactory receptor neurons, as described previously (Madhwal et al., 2020; Shim et al., 2013), by expressing the pro-apoptotic gene *hid* in all olfactory neurons using *Orco-Gal4* as the driver. We observed that this genetic manipulation led to a stark reduction in lymph gland size (Fig. 6B) with a dramatic increase in their ROS levels (Fig. 6C-E). We also observed that olfactory dysfunction (*Orco>hid*) led to a significant reduction in the levels of pPDH (Fig. 6F,G,J) and pPDK (Fig. 6H,I,K) in lymph glands, without altering total PDH or PDK (Fig. S6O,P). These data were similar to progenitor *Gat* and *Ssadh* loss of function, and suggested that loss of olfaction via a reduction in systemic GABA levels led to dysregulation of lymph gland progenitor-cell PDK activity. This consequently raised their TCA rate, leading to increased ROS generation that negatively impacted lymph gland growth. In summary, these data show that, during homeostasis, hematopoietic growth and immune-progenitor redox balance is sensitive to animal odor-sensing and its systemic axis of regulation.

## DISCUSSION

**Fig. 7.**
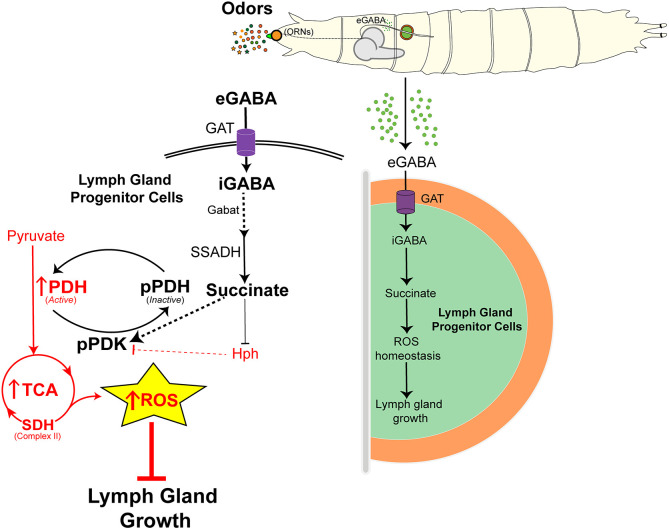
**Olfaction-derived systemic GABA in lymph gland ROS homeostasis and growth control.** The model describes the importance of olfaction-derived GABA metabolism in lymph gland growth control. All elements that repress growth are shown in red, while positive regulators of growth are shown in black. Blood progenitor cells of the *Drosophila* larval lymph gland maintain ROS in them that is derived from the TCA cycle. However, heightened or uncontrolled TCA cycle activity, leading to increased ROS production in progenitor cells, abrogates lymph gland growth and development. Thus, to moderate progenitor TCA activity and ROS levels, *Drosophila* larvae rely on olfaction-derived systemic GABA (eGABA). Sensing extracellular GABA (eGABA) via blood progenitor cells and its subsequent metabolism through the GABA catabolic pathway into succinate promotes the maintenance of pPDK, which is the active form of PDK. PDK phosphorylates PDH (pPDH) and inactivates it. PDH is a key rate-limiting enzyme of the TCA cycle, the activation of which drives TCA cycle activity. Thus, GABA metabolism in progenitor cells via activating PDK limits the TCA cycle and subsequently maintains ROS homeostasis therein. This metabolic state supports normal lymph gland growth and development. In the absence of any olfactory input or if the use of GABA by blood progenitors is lost, the lack of succinate in progenitor cells leads to increased Hph function, which blocks PDK activation that consequently leads to heightened TCA cycle activity and ROS generation. This leads to lymph gland growth retardation.

In this study, we show that blood progenitor cells of the lymph gland rely on the TCA cycle and OXPHOS to generate intracellular ROS. Although physiological levels of ROS do not control growth, their increased generation leads to retardation of lymph gland growth. Therefore, to control ROS production, the progenitor cells internalize olfaction-derived systemic GABA and, via its breakdown into succinate, the cells activate PDK function. This facilitates PDH phosphorylation, which limits TCA cycle activity and consequently ROS production, and supports lymph gland growth. In conditions with low progenitor GABA metabolism, the lack of succinate generation from this pathway promotes Hph function and a reduction in PDK activation. This consequently leads to heightened TCA cycle activity, increased ROS production and abrogation of lymph gland growth. Thus, conditions leading to a block in progenitor GABA metabolism are susceptible to alterations in redox balance and subsequently hematopoietic growth defects. In this regard, animals with olfactory dysfunction that show a reduction in systemic GABA (Shim et al., 2013) have heightened ROS and smaller lymph glands. Taken together, we propose that *Drosophila* larvae rely on GABA derived from environmental odor-sensing as a means to moderate blood progenitor TCA cycle activity and ROS balance to maintain normal lymph gland growth and development (Fig. 7).

### Regulators of blood-progenitor ROS

The central theme of the work undertaken in this study was based on the understanding that ROS as a signaling entity are critical for blood stem progenitor development and maintenance, as reported both in invertebrates and vertebrates (Bigarella et al., 2014; Harris et al., 2013; Prieto-Bermejo et al., 2018; Takubo et al., 2013; Tothova et al., 2007; Vincent and Crozatier, 2010). However, to sustain this developmental role, mechanisms controlling ROS levels that are crucial for its functioning in myeloid progenitor cells remain fairly uncharacterized. We show the importance of TCA activity and OXPHOS in the generation of ROS in progenitor cells during homeostasis and the importance of pyruvate oxidation driving TCA activity. Loss of PDK function data proves PDK importance in the overall growth of the blood tissue. However, loss of either *Pdha* or *SdhA* function in progenitor cells, which resulted in further lowering of TCA and OXPHOS, failed to show any lymph gland growth phenotype. This implied that physiological levels of ROS did not contribute to lymph gland growth; it is only when ROS levels were higher than the basal threshold that it produced a growth defect. The independence of physiological ROS levels from growth control is intriguing but needs a more thorough investigation. Additional sources of ROS, such as Duox- and NOx-dependent mechanisms (Rada and Leto, 2008) or TCA cycle-derived succinate in reverse electron transport (RET) (Chouchani et al., 2014) are possibilities that have not been addressed.

The regulation of ROS by catabolism of GABA-derived succinate, as opposed to TCA-derived succinate (which drives ROS generation), also reveals that spatial localization and the availability of metabolites are distinct regulators of intracellular outcomes. Like the TCA cycle, GABA breakdown also takes place in the mitochondria, but our recent data (Madhwal et al., 2020) and the current work highlight a distinct role for GABA-derived succinate in controlling cytosolic functions such as Sima stabilization (Madhwal et al., 2020) and PDK enzyme activity. This raises the question of why TCA cycle-derived succinate is not available to perform functions conducted by GABA-derived succinate? The TCA cycle rate may be a limiting factor or the two pathways could contribute to spatially distinct pools of succinate that perform different functions. However, this theory remains speculative, and metabolic flux analysis and spatial resolution of metabolites at a subcellular level will be needed to address it.

### GABA in myeloid development

Recent findings have showcased the importance of GABA in myeloid immunity specifically with its role in metabolic programming of myeloid cells during innate immune training (Shao et al., 2021; Steidl et al., 2004; Zhu et al., 2019). These studies indicate commonalities between the myeloid system of mammals and *Drosophila*. Our findings in the *Drosophila* hematopoietic system highlight the multiple developmental roles performed by GABA in myeloid development, progenitor homeostasis and immunity. The underlying crosstalk of GABA with other pathways to moderate such diverse functions (Madhwal et al., 2020; Shim et al., 2013) is also apparent. In homeostasis, the role of GABA as a ligand to activate GABA_B_R signaling and regulation of intracellular calcium homeostasis in progenitor cells maintains these cells in their undifferentiated state (Shim et al., 2013). In the context of immunity, GABA, via its metabolism, inhibits Hph and promotes Hifα/Sima stabilization to support a successful immune response (Madhwal et al., 2020). In this study, we show that GABA is a necessary metabolite for hematopoietic growth. Although the loss of GABA breakdown leads to hematopoietic growth retardation, its increase in blood cells is accompanied by a concomitant increase in lymph gland size. The regulation of PDK by GABA metabolism maintains homeostatic levels of progenitor ROS that support normal lymph gland growth. The growth advantage provided by increased GABA, as seen in *Gat* overexpression, is via a TCA cycle and/or ROS-independent pathway that remains to be understood. In this study, we further find that GABA-mediated regulation of TCA activity is also necessary for proper lamellocyte induction during immune challenge. Together, the data reveal that, in addition to promoting Sima (Madhwal et al., 2020), GABA, via PDK, inhibits TCA to bring about a successful immune response. Thus, GABA in *Drosophila* myeloid progenitor cells functions at the nexus of coordinating multiple intracellular signaling and metabolic events that define cell fate decisions, and GABA emerges as a central regulator of myeloid development and function.

### Conclusions

Olfaction is a key stress-sensing sensory modality in animals (Ache and Young, 2005; Su et al., 2009) and strikingly, a strong olfactory/immune connect is seen across systems (Strous and Shoenfeld, 2006; Madhwal et al., 2020; Shim et al., 2013). In this study, we show the regulation of blood-progenitor redox balance and lymph gland growth by olfaction. We have previously reported the importance of olfaction-axis in immune priming and the generation of successful immune responses. We therefore speculate the sensory module may have evolved to engage with the immune system to modulate its development as per environmental demands. Depending on the physiological context, environmental odor-sensing relays information to immune-progenitor cells to moderate their development or differentiation accordingly. Overall, a model describing the olfaction-mediated GABA-dependent regulation of progenitor redox balance and growth is proposed in Fig. 7. The results demonstrate that myeloid metabolism and ROS balance in them is sensitive to olfaction-derived GABA. This axis is necessary to maintain proper lymph gland growth and development. Whether the findings presented here are relevant for the development of blood cells in higher organisms with complex lineages remains to be tested.

## MATERIALS AND METHODS

### *Drosophila* husbandry, stocks and genetics

The following *Drosophila melanogaster* stocks were used in this study: *w^1118^*, *domeMeso-Gal4, UAS-GFP* and *TepIV-Gal4, UAS-mCherry* (Banerjee lab, UCLA, USA); *Hml^Δ^-Gal4, UAS-2xEGFP* (S. Sinenko, Russian Academy of Sciences, Moscow); *Orco-Gal4* (BDSC 26818); *UAS-Hph* (B. Edgar, Huntsman Cancer Institute, University of Utah, Salt Lake City, USA; ETH Zürich, Switzerland); and *UAS-Gat* (M. Freeman, Vollum Institute, Oregon Health & Science University, Portland, USA). The RNAi stocks were obtained from the VDRC and the BDSC *Drosophila* stock centers. The lines used in this study are *Gat^RNAi^* (BL29422), *Ssadh^RNAi^* (v106637, BL55683), *SdhA^RNAi^* (v330053), *Hph^RNAi^* (v103382), *Pdha^RNAi^* (BL55345), *Pdk^RNAi^* (BL28635, BL 35142), *Catalase^RNAi^* (*Cat^RNAi^*, BL43197), *Sod2^RNAi^* (BL 24489), *UAS-Catalase* (*Cat*, BL24621) and *UAS-hid* (BL65403)*.* All fly stocks were reared on cornmeal agar food medium with yeast supplementation in 25°C incubators unless otherwise specified. Tight collections were carried out for 4-6 h to avoid overcrowding and for synchronous development of larvae. The crosses involving RNAi lines were maintained at 29°C to maximize the efficacy of the *Gal4/UAS* RNAi system. Controls correspond to Gal4 driver crossed with *w^1118^* or *w^1118/1118^*.

### ROS (DHE) detection in lymph glands

Lymph glands dissected from the wandering 3rd instar larvae were stained for ROS levels following the protocol of Owusu-Ansah et al. (2008). The dissected lymph gland tissues were stained in 1:1000 DHE (Invitrogen, Molecular Probes, D11347) dissolved in 1×PBS for 15 min in the dark. Tissues were washed in 1×PBS twice and fixed with 4% formaldehyde for 6-8 min at room temperature in the dark. Tissues were again quickly washed in 1×PBS twice and then mounted in Vectashield (Vector Laboratories). The lymph glands were imaged immediately. A minimum of five samples was analyzed per experiment and each experiment was repeated at least three times. One representative image (one lymph gland lobe) is shown in the figure panels.

### Immunostaining and immunohistochemistry

Immunohistochemistry on lymph gland tissues were performed with the following primary antibodies: mouse-αP1 (I. Ando, Hungarian Academy of Sciences, Szeged, Hungary; 1:30), rabbit-αPPO (1:1000; H. M. Müller, EMBL, Heidelberg, Germany), mouse-αMys (1:100, DSHB, CF.6G11), mouse-αPDH (Abcam, ab110334, 1:250), mouse-αPDK (Abcam, ab110025, 1:500), rabbit-αpPDH (Abcam, S293, ab177461, 1:250), rabbit-αpPDK (Signalway Antibody TYR243, SAB #11597, 1:100) and rabbit-αGat (1:2000, M. Freeman Lab). The secondary antibodies Alexa Fluor 488, 546 and 647 (Invitrogen) were used at 1:400; phalloidin (Invitrogen) was used at 1:100. Nuclei were visualized using DAPI (Sigma). Samples were mounted with Vectashield (Vector Laboratories).

Lymph glands dissected from wandering 3rd instar larvae were stained following the protocol of Jung et al. (2005). Lymph gland tissues from synchronized larvae of the required developmental stage were dissected in ice-cold PBS (pH 7.2) and fixed in 4% formaldehyde solution for 40 min at room temperature. Tissues were then washed thrice (15 min per wash) in 0.3% PBT (0.3% Triton-X in 1×PBS) for permeabilization and were further blocked in 5% normal goat serum (NGS, Jackson ImmunoResearch, 005-000-121), for 45 min at room temperature. Tissues were next incubated in the respective primary antibodies with appropriate dilution in 5% NGS overnight at 4°C. After primary antibody incubation, tissues were washed thrice in 0.3% PBT for 15 min each. This was followed by incubation of tissues in respective secondary antibodies for 2-3 h at room temperature. After secondary antibody incubation, tissues were washed in 0.3% PBT for 15 min following a DAPI+0.3% PBT wash for 15 min. Excess DAPI was washed off by a wash of 0.3% PBT for 15 min. Tissues were mounted in Vectashield (Vector Laboratories) and then imaged using confocal microscopy (Olympus FV3000). A minimum of five were analyzed per experiment and each experiment was repeated at least three times. One representative image (one lymph gland lobe) is shown in the figure panels.

### Image acquisition and processing

DHE stained (ROS) and immunostained lymph gland tissues images were acquired using Olympus FV3000 Confocal Microscopy 40× oil-immersion objective. Microscope settings were kept constant for each sample in every experiment. Specifically, for ROS, the image acquisition settings were chosen to capture the difference between MZ and CZ ROS levels. The medullary zone containing the blood progenitor cells has elevated ROS compared with the differentiating cells of the cortical zone (Owusu-Ansah and Banerjee, 2009). The image acquisition settings were chosen to capture this difference in control lymph glands without causing saturation in the majority of the pixels. This setting was thereafter kept constant for all other genotypes that were conducted in the corresponding experimental batch and were processed for analysis and quantifications. Lymph gland images were processed using ImageJ (NIH) and Adobe Photoshop CS5 software.

Specifically, image files were processed using Photoshop. The primary image was resized along with the scale bar and then one lobe of the lymph gland was cropped for representation in the figure panels. The background that is generally present around the lymph gland lobe (e.g. tissues such as the ring gland, brain, discs and dorsal vessel) was removed using the erase tool and the lymph gland lobe was outlined with a white dotted line using the pencil tool. This image is shown as the representative image in the figure panels. This procedure was carried out for all lymph gland images and no additional processing or manipulations were carried out on the images.

### Quantification of lymph gland phenotypes

All images were quantified using ImageJ (NIH) software and Microsoft Excel. Images were acquired as *z*-stacks and quantifications were carried out as described previously (Shim et al., 2012). For lymph gland area analysis, the middle two *z*-stacks were merged, and the total lymph gland area was marked using the free-hand tool of ImageJ and then analyzed for quantifications. The relative fold change in ROS levels and the intensities per lobe was calculated using mean fluorescence intensity values. ROS quantifications were carried out from the entire lymph gland lobe. In the genetic backgrounds where progenitor-specific knockdown was carried out, intensity quantifications were carried out only from the dome^+^ (blood-progenitor cells) area; in other backgrounds, the entire lymph gland lobe was marked and then quantified for mean fluorescence intensity. Background noise was quantified from the unmarked zones at four random regions (marked by equal-sized square boxes) and subtracted from the mean intensity values. The relative fold change was calculated from the final mean fluorescence intensity values in Microsoft Excel and graph plotting; statistical data analysis was performed using GraphPad Prism software. For all intensity quantifications, the laser settings for each experimental set-up were kept constant and controls were analyzed in parallel with the mutant conditions every time.

### Lymph gland differentiation analysis

A single middle stack image was obtained from each lymph gland lobe from which areas of the respective populations were obtained by using the freehand tool on ImageJ to select areas in the respective channels. The images were marked accordingly to extract total lymph gland area (DAPI^+^ channel, C1), total dome^+^ area (Dome-GFP^+^ channel only, C2), total P1^+^ area (P1 channel only, C3), and total area covering all dome^+^ and P1^+^ regions (Dome-GFP^+^ channel merge with P1channel, C4). From here, the percentage of Dome^+^P1^+^ double-positive cells was obtained by subtracting the area of all dome^+^ and P1^+^regions (C4) with combined areas from the individually marked Dome^+^ (C2) and P1^+^ (C3) regions. This was then represented as the percentage with respect to total lymph gland area [C1, Dome^+^P1^+^ double-positive=(C2+C3)−C4/C1*100]. To calculate percentage Dome^−^P1^−^ area, dome^+^P1^+^ area (C4) was subtracted from total lymph gland area (C1, Dome^−^P1^−^=C1−C4/C1*100).

### Wasp culture and infection

*Leptopilina boulardi* wasps were maintained as previously described (Schlenke et al., 2007). For wasp infections, the protocol of Bajgar et al. (2015) was followed. Early 3rd instar larvae were exposed to 10-15 females and 5-8 male *L. boulardi* wasps for 6 h at 25°C. After removing wasps, the infected *Drosophila* larvae were kept back to 29°C until respective analyses, i.e. 24 h for lymph gland lamellocyte analyses and 48 h for circulation lamellocyte analyses.

### Crystal cells and lamellocytes count

For crystal cells analysis, lymph glands were stained with PPO1 to mark the crystal cells. The stained tissues were then imaged and *z*-stacks were acquired. Crystal cells were counted manually from entire *z*-stacks per lymph gland lobe using ImageJ.

For lamellocyte count at 24 hours post-infection (HPI), the lymph gland tissues were stained with Myospheroid to mark the lamellocytes and imaged to obtain *z*-stacks. Lamellocytes were then manually counted as carried out for crystal cells. For circulating lamellocytes count at 48 HPI, individual larvae were bled on Teflon-printed microscopic slides (Immuno-Cell International, one larva per well), then counterstained with phalloidin; lamellocytes were counted manually based on their large flattened morphology under the microscope, as described previously (Madhwal et al., 2020).

### Metabolite supplementation

Succinate (sodium succinate dibasic hexahydrate, Sigma, S9637) and N-Acetyl-L-cysteine (NAC, Sigma, A7250) enriched diets were prepared by supplementing regular fly food with weight/volume measures of succinate and NAC to achieve 3% and 0.1% concentrations, respectively. Eggs were transferred in these supplemented diets and reared until analysis of the respective tissues (lymph gland).

### Sample size and statistical analyses

In all experiments, *n* implies the total number of samples analyzed that were obtained from multiple independent experimental repeats and ‘*N*’ represents the number of independent experimental repeats, which is shown by green dot in the graphs. All experiments have been repeated a minimum of three times; in each experimental setup, at least 5-10 animals were analyzed. *Drosophila* availability is not limiting; therefore, no power calculations were used to predetermine sample size.

All statistical analyses and quantifications were performed using GraphPad Prism Nine and Microsoft Excel 2016. In box and whisker plots, the horizontal line indicates the median, whiskers indicate the minimum and maximum values, and the box indicates the lower and upper quartiles. Two-way ANOVA with Tukey's post-hoc test is employed for multiple comparisons to account for the variation between and within the experiments (Hadjieconomou et al., 2020). A two-way ANOVA Dunnett's multiple comparison test was used for differentiation analysis; graphs are plotted as mean±s.d.

## Supplementary Material

Supplementary information

Reviewer comments
