# Supplementary material for: Blood progenitor redox homeostasis through olfaction-derived systemic GABA in hematopoietic growth control in Drosophila
Source: Development. 2021 Dec 1;149(8):dev199550. doi: 10.1242/dev.199550 (PMC8733872; doi:10.1242/dev.199550)
Supplement: Supplementary information [file develop-149-199550-s1.pdf]

Fig. S1

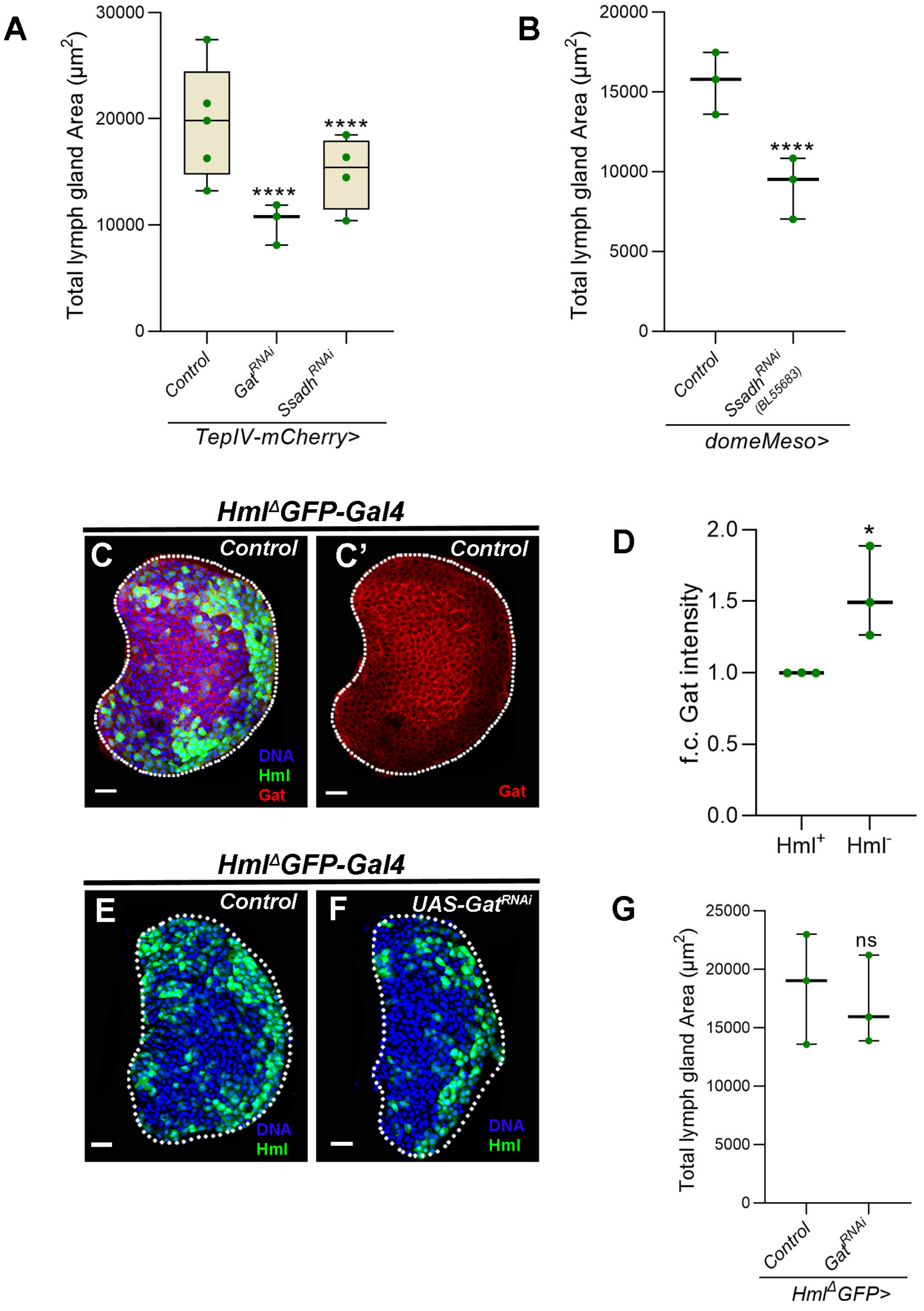

Development • Supplementary information

Fig. S1. GABA catabolism in *Drosophila* blood progenitor cells control lymph gland growth.

(A) Quantifications of lymph gland size in *TepIV-Gal4;UAS-mCherry/+* (control, N=5, n=40), *TepIV-Gal4;UAS-mCherry;UAS-Gat^{RNAi}* (N=3, n=32, p<0.0001) and *TepIV-Gal4;UAS-mCherry;UAS-Ssadh^{RNAi}* (N=4, n=30, p<0.0001).

(B) Quantifications of lymph gland size in *domeMeso>GFP/+* (control, N=3, n=30) and *domeMeso>GFP/Ssadh^{RNAi}* $^{(BL55683)}$ (N=3, n=30, p<0.0001).

(C,C') Representative images showing Gat protein expression in control lymph gland (*Hml$^{\Delta}$-Gal4;UAS-GFP/+*), **(C)** with Hml^{+} overlap (green) and **(C')** without Hml^{+} overlap, Hml^{+} region show lesser Gat levels as compared to Hml^{-}. For quantifications, refer to **D.**

(D) Relative fold change in Gat levels in *Hml$^{\Delta}$GFP>/+* in the Hml^{+} (N=3, n=16) and Hml^{-} region (N=3, n=16, p=0.0153) of the lymph gland.

(E-F) Representative images showing lymph gland size in differentiating cells specific (Hml^{+}) loss of *Gat*, **(F)** expressing *Gat^{RNAi}* (*Hml$^{\Delta}$-Gal4,UAS-GFP;UAS-Gat^{RNAi}*) does not show any reduction in lymph gland size as compared to **(E)** control (*Hml$^{\Delta}$-Gal4;UAS-GFP/+*). For quantifications, refer to **G**.

(G) Quantifications of lymph gland size in *Hml$^{\Delta}$GFP>/+* (control, N=3, n=34) and *Hml$^{\Delta}$GFP>/Gat^{RNAi}* (N=3, n=30, p=0.1921).

Data is presented as median plots (*p<0.05;**p<0.01;***p<0.001,****p<0.0001,n.s.=non-significant), two-way ANOVA, Tukey's multiple comparisons test. f.c.= fold change. Scale bar: 20µm.'n'=lymph gland lobes. 'N'= number of experimental repeats (green dot). DAPI marks DNA. Lymph gland lobes are outlined with a white border and for clarity purposes the accompanying background containing other tissues, such as ring gland, brain, dorsal vessel, etc., has been removed.

Development • Supplementary information

Fig. S2

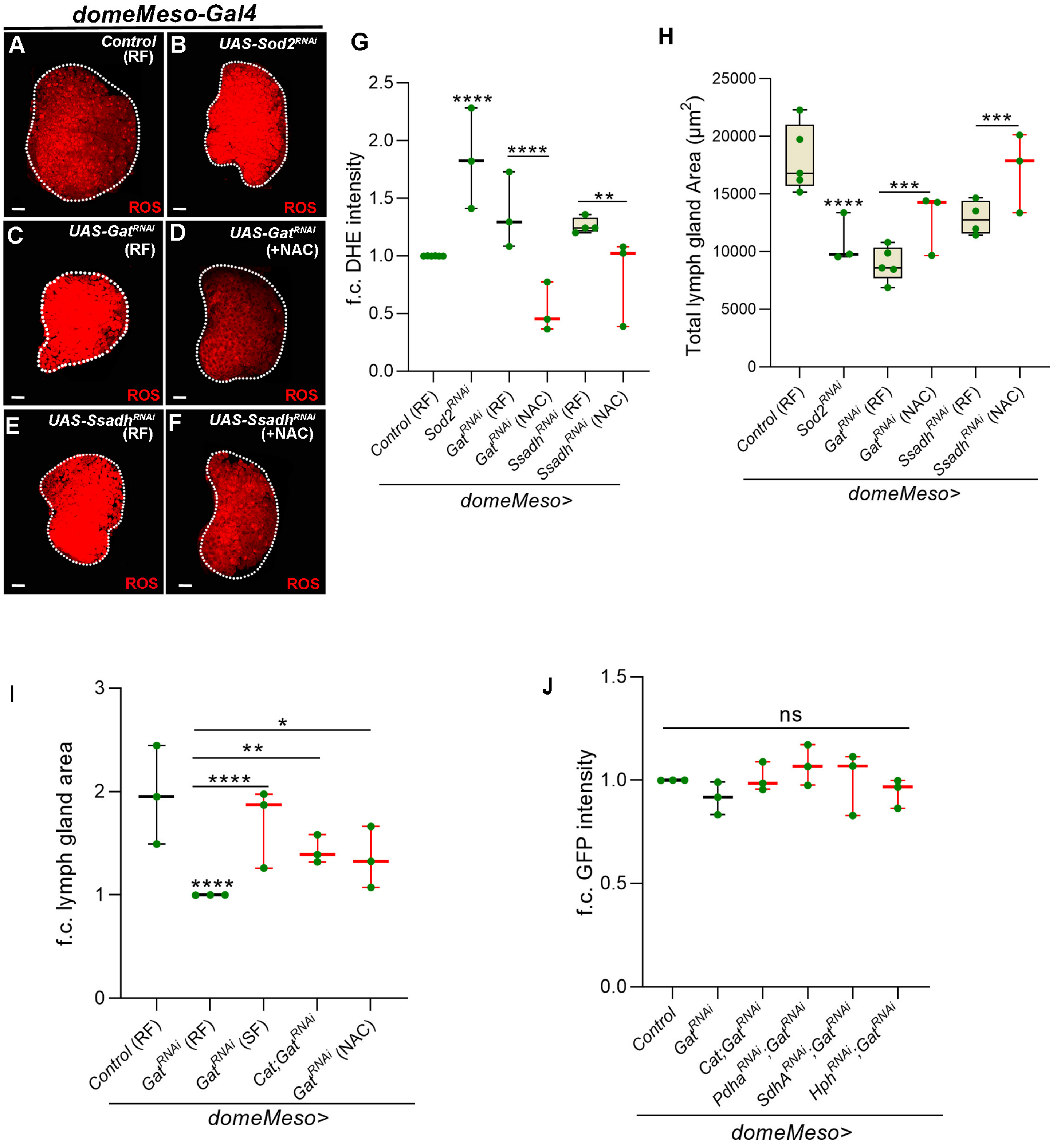

Development • Supplementary information

Fig. S2. ROS regulation by GABA shunt pathway in *Drosophila* blood progenitors is important for lymph gland growth.

(A-F) Representative lymph gland images showing ROS levels, **(A)** control (*domeMeso-Gal4,UAS-GFP/+*) lymph gland showing higher ROS levels in the blood progenitor cells, **(B)** expressing *Sod2^{RNAi}* (*domeMeso-Gal4,UAS-GFP;UAS-Sod2^{RNAi}*) in blood progenitor cells leads to increase in ROS levels as compared to **(A)** control, **(C-F)** NAC supplementation to (**D)** *domeMeso-Gal4,UAS-GFP;UAS-Gat^{RNAi}* and **(F)** *domeMeso-Gal4,UAS-GFP;UAS-Ssadh^{RNAi}* rescues the increased ROS phenotype as compared to **(C)** *domeMeso-Gal4,UAS-GFP;UAS-Gat^{RNAi}* and **(E)** *domeMeso-Gal4,UAS-GFP;UAS-Ssadh^{RNAi}* on RF respectively. For quantifications, refer to **G**.

(G) Relative fold change in lymph gland ROS (DHE) levels in *domeMeso>GFP*/+ (control, N=6, n=49), *domeMeso>GFP/Sod2^{RNAi}* (N=3, n=10, p<0.0001), *domeMeso>GFP/Gat^{RNAi}* (RF, N=3, n=24, p=0.0092), *domeMeso>GFP/Gat^{RNAi}* (NAC, N=3, n=28, p<0.0001), *domeMeso>GFP/Ssadh^{RNAi}* (RF, N=4, n=23, p=0.0431) and *domeMeso>GFP/Ssadh^{RNAi}* (NAC, N=3, n=24, p=0.0019).

(H) Quantifications of lymph gland area in *domeMeso>GFP*/+ (control, N=5, n=37), *domeMeso>GFP/Sod2^{RNAi}* (N=3, n=40, p<0.0001), *domeMeso>GFP/Gat^{RNAi}* (RF, N=5, n=42, p<0.0001), *domeMeso>GFP/Gat^{RNAi}* (NAC, N=3, n=26, p=0.0003), *domeMeso>GFP/Ssadh^{RNAi}* (RF, N=4, n=35, p<0.0001) and *domeMeso>GFP/Ssadh^{RNAi}* (NAC, N=3, n=20, p=0.0001).

(I) Relative fold change in lymph gland area in *domeMeso>GFP* /+ (control, N=3, n=26), *domeMeso>GFP/Gat^{RNAi}* (RF, N=3, n=26, p<0.0001 compared to control), *domeMeso>GFP/Gat^{RNAi}* (SF, N=3, n=26, p<0.0001), *domeMeso>GFP/Cat;Gat^{RNAi}* (N=3, n=26, p=0.0079) and *domeMeso>GFP/Gat^{RNAi}* (NAC, N=3, n=26, p=0.0162) compared to *domeMeso>GFP/Gat^{RNAi}*.

(J) Relative fold change in dome^{+} GFP intensity in *domeMeso>GFP* /+(control, N=3, n=23), *domeMeso>GFP/Gat^{RNAi}* (RF, N=3, n=19, n=0.8701), *domeMeso>GFP/Cat;Gat^{RNAi}* (N=3, n=19, p>0.9999), *domeMeso>GFP/Pdha^{RNAi};Gat^{RNAi}* (N=3, n=20, p=0.7488), *domeMeso>GFP/SdhA^{RNAi};Gat^{RNAi}* (N=3, n=18, p>0.9999) and *domeMeso>GFP/Hph^{RNAi};Gat^{RNAi}* (N=3, n=21, p=0.9565).

Development • Supplementary information

RF is regular food, SF is succinate food and NAC is N-acetylcysteine supplemented food. Data is presented as median plots (*p<0.05;**p<0.01;***p<0.001,****p<0.0001,n.s.=non-significant), two-way ANOVA, Tukey's multiple comparisons test. f.c.= fold change. Scale bar: 20µm.'n'=lymph gland lobes. 'N'= number of experimental repeats (green dot). DAPI marks DNA. Comparisons for significance are with control values, unless marked by horizontal lines for other respective comparisons and red bars represent rescue combinations. Lymph gland lobes are outlined with a white border and for clarity purposes the accompanying background containing other tissues, such as ring gland, brain, dorsal vessel, etc., has been removed.

Fig. S3

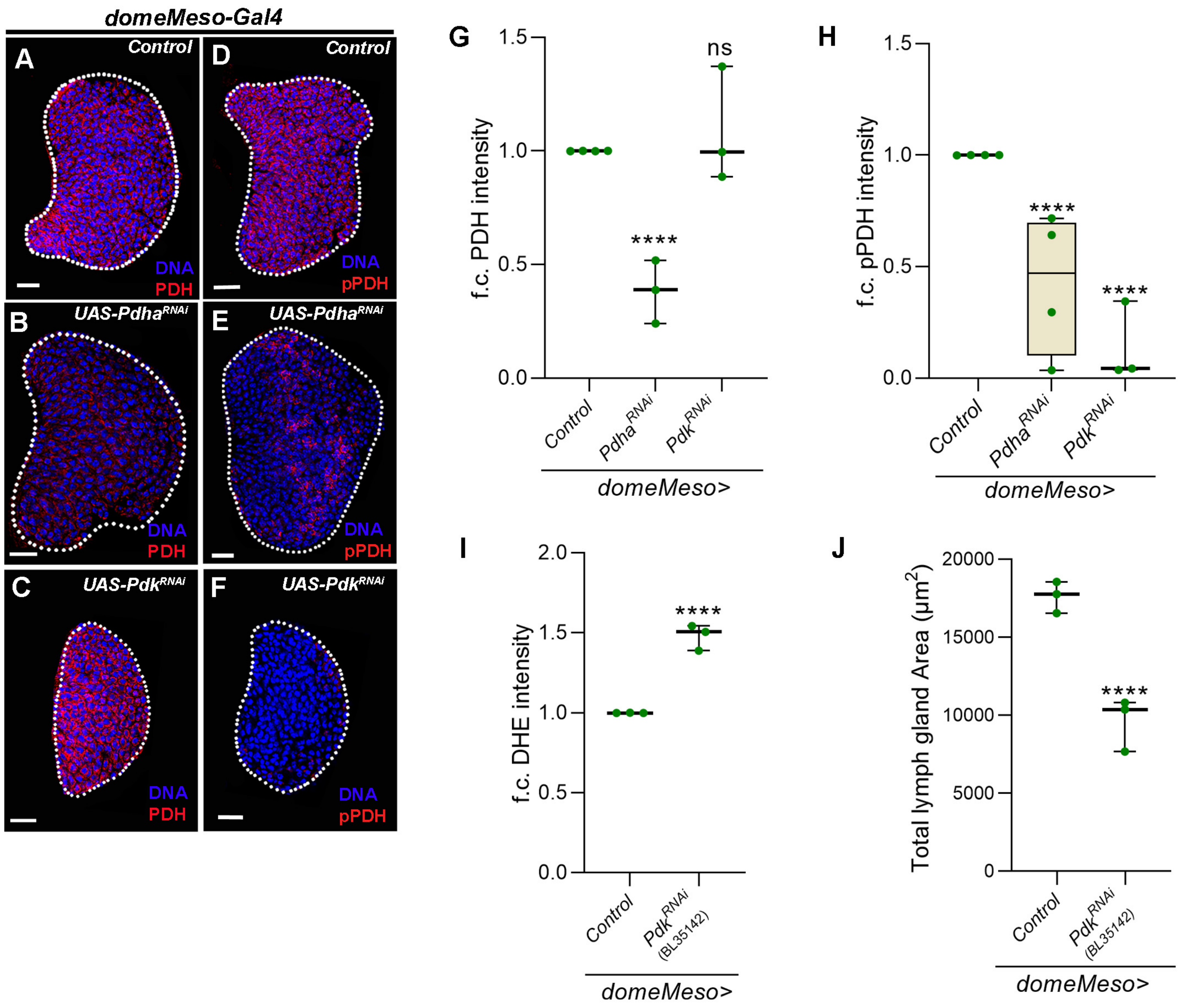

Development • Supplementary information

Fig. S3. TCA activity regulates blood-progenitor ROS levels and lymph gland growth.

(A-C) Representative lymph gland images showing PDH (red), **(A)** control (*domeMeso-Gal4,UAS-GFP/+*)**, (B)** expressing *Pdha*^{RNAi} in progenitor cells (*domeMeso-Gal4,UAS-GFP;UAS-Pdha*^{RNAi}) show reduction in medullary zone PDH levels and **(C)** expressing *Pdk*^{RNAi} in progenitor cells (*domeMeso-Gal4,UAS-GFP;UAS-Pdk*^{RNAi}) does not show reduction in medullary zone PDH levels as compared to **(A)** control**.** For quantifications, refer to **G.**

(D-F) Representative lymph gland images showing pPDH (red), **(A)** control (*domeMeso-Gal4,UAS-GFP/+*)**, (B)** expressing *Pdha*^{RNAi} in progenitor cells (*domeMeso-Gal4,UAS-GFP;UAS-Pdha*^{RNAi}) show reduction in medullary zone pPDH levels and **(C)** expressing *Pdk*^{RNAi} in progenitor cells (*domeMeso-Gal4,UAS-GFP;UAS-Pdk*^{RNAi}) also show reduction in medullary zone pPDH levels as compared to **(D)** control**.** For quantifications, refer to **H.**

(G-H) Relative fold change in lymph gland MZ **(G)** PDH levels in *domeMeso>GFP/+* (control, N=4, n=41), *domeMeso>GFP/Pdha*^{RNAi} (N=3, n=29, p<0.0001) and *domeMeso>GFP/Pdk*^{RNAi} (N=3, n=33, p=0.9240) and **(H)** pPDH levels in *domeMeso>GFP*/+ (control, N=4, n=49), *domeMeso>GFP*/*Pdha*^{RNAi} (N=4, n=38, p<0.0001), and *domeMeso>GFP/Pdk*^{RNAi} (N=3, n=48, p<0.0001).

(I) Relative fold change in lymph gland ROS (DHE) levels in *domeMeso>GFP*/+ (control, N=3, n=37), and *domeMeso>GFP*/*Pdk*$^{RNAi\ (BL35142)}$ (N=3, n=31, p<0.0001).

(J) Quantifications of lymph gland area in *domeMeso>GFP*/+ (control, N=3, n=30), and *domeMeso>GFP/Pdk*$^{RNAi\ (BL35142)}$ (N=3, n=30, p<0.0001).

Data is presented as median plots (*p<0.05;**p<0.01;***p<0.001,****p<0.0001,n.s.=non-significant), two-way ANOVA, Tukey's multiple comparisons test. f.c.= fold change. MZ=Medullary Zone. Scale bar: 20µm.'n'=lymph gland lobes. 'N'= number of experimental repeats (green dot). DAPI marks DNA. Lymph gland lobes are outlined with a white border and for clarity purposes the accompanying background containing other tissues, such as ring gland, brain, dorsal vessel, etc., has been removed.

Fig. S4

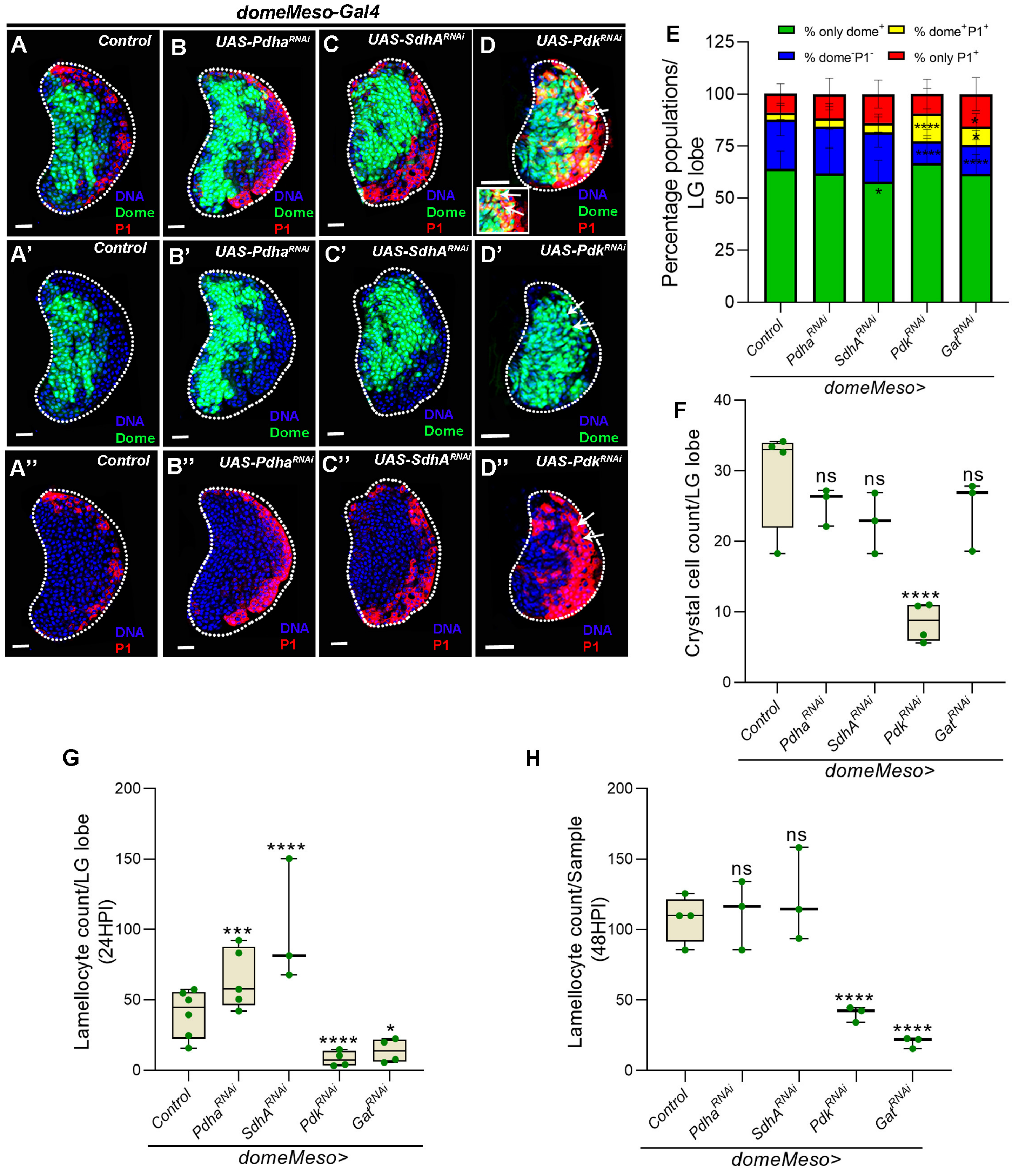

Development • Supplementary information

Fig. S4. Modulations in TCA activity affects blood progenitor differentiation in homeostasis and immune response upon wasp-infection.

(A-D'') Representative images showing lymph gland growth and differentiation status, **(A-A'')** control (*domeMeso-Gal4,UAS-GFP*/+) showing **(A)** dome^{+} (green) and P1^{+} (red), **(A')** dome^{+}(green) and **(A'')** P1^{+} (red), **(B-B'')** expressing *Pdha^{RNAi}* (*domeMeso-Gal4,UAS-GFP;UAS-Pdha^{RNAi}*) and **(C-C'')** expressing *SdhA^{RNAi}* (*domeMeso-Gal4,UAS-GFP;UAS-SdhA^{RNAi}*) does not show any major defect in lymph gland differentiation status, expressing **(D-D'')** *Pdk^{RNAi}* in progenitor cells (*domeMeso-Gal4,UAS-GFP;UAS-Pdk^{RNAi}*) leads to small lymph gland size and appearance of **(D)** dome^{+}p1^{+} overlap population (shown in inset and by white arrows). For quantifications, refer to **E**.

(E) Quantifications of lymph gland differentiation status shown as percentage of only dome^{+}(green), dome^{-}P1^{-}(blue), dome^{+}P1^{+}(yellow) and only P1^{+}(red) populations per lymph gland lobe. p-values are presented in the preceding order. *domeMeso>GFP*/+(control, n=30), *domeMeso>GFP/Pdha^{RNAi}* (n=30, p=0.6970, 0.9647, 0.9976, 0.5921), *domeMeso>GFP/SdhA^{RNAi}* (n=30, p=0.0135, 0.9999, 0.9649, 0.0890), *domeMeso>GFP/Pdk^{RNAi}* (n=30, p=0.5046, <0.0001, <0.0001, 0.9999) and *domeMeso>GFP/Gat^{RNAi}* (n=26, p=0.6528, <0.0001, 0.0456, 0.0139).

(F) Quantifications of crystal cell count per lymph gland lobe in *domeMeso>GFP*/+ (control, N=4, n=63), *domeMeso>GFP/Pdha^{RNAi}* (N=3, n=32, p=0.8486), *domeMeso>GFP/Pdk^{RNAi}* (N=4, n=45, p<0.0001), *domeMeso>GFP/SdhA^{RNAi}* (N=3, n=44, p=0.2574) and *domeMeso>GFP/Gat^{RNAi}* (N=3, n=30, p=0.341).

(G) Quantifications of lamellocyte count per lymph gland lobe at 24HPI in *domeMeso>GFP*/+ (control, N=6, n=66), *domeMeso>GFP/Pdha^{RNAi}* (N=5, n=60, p=0.0002), *domeMeso>GFP/Pdk^{RNAi}* (N=4, n=43, p=0.0002), *domeMeso>GFP/SdhA^{RNAi}* (N=3, n=11, p<0.0001) and *domeMeso>GFP/Gat^{RNAi}* (N=4, n=46, p=0.0167).

(H) Quantifications of lamellocyte count in circulation at 48HPI in *domeMeso>GFP*/+ (control, N=4, n=54), *domeMeso>GFP/Pdha^{RNAi}* (N=3, n=30, p=0.9948), *domeMeso>GFP/Pdk^{RNAi}* (N=3, n=71, p<0.0001), *domeMeso>GFP/SdhA^{RNAi}* (N=3, n=22, p=0.9435) and *domeMeso>GFP/Gat^{RNAi}* (N=3, n=22, p<0.0001).

Data is presented as median plots (*p<0.05;**p<0.01;***p<0.001,****p<0.0001,n.s.=non-significant), two-way ANOVA, Tukey's multiple comparisons test and Dunnett's multiple comparison test for **E** (mean±SD). f.c.= fold change. MZ=Medullary Zone. Scale bar: 20µm.'n'=lymph gland lobes and number of animals analysed for **H**. 'N'= number of experimental repeats (green dot). DAPI marks DNA. HPI indicates hours post-infection. Lymph gland lobes are outlined with a white border and for clarity purposes the accompanying background containing other tissues, such as ring gland, brain, dorsal vessel, etc., has been removed.

Development • Supplementary information

Fig. S5

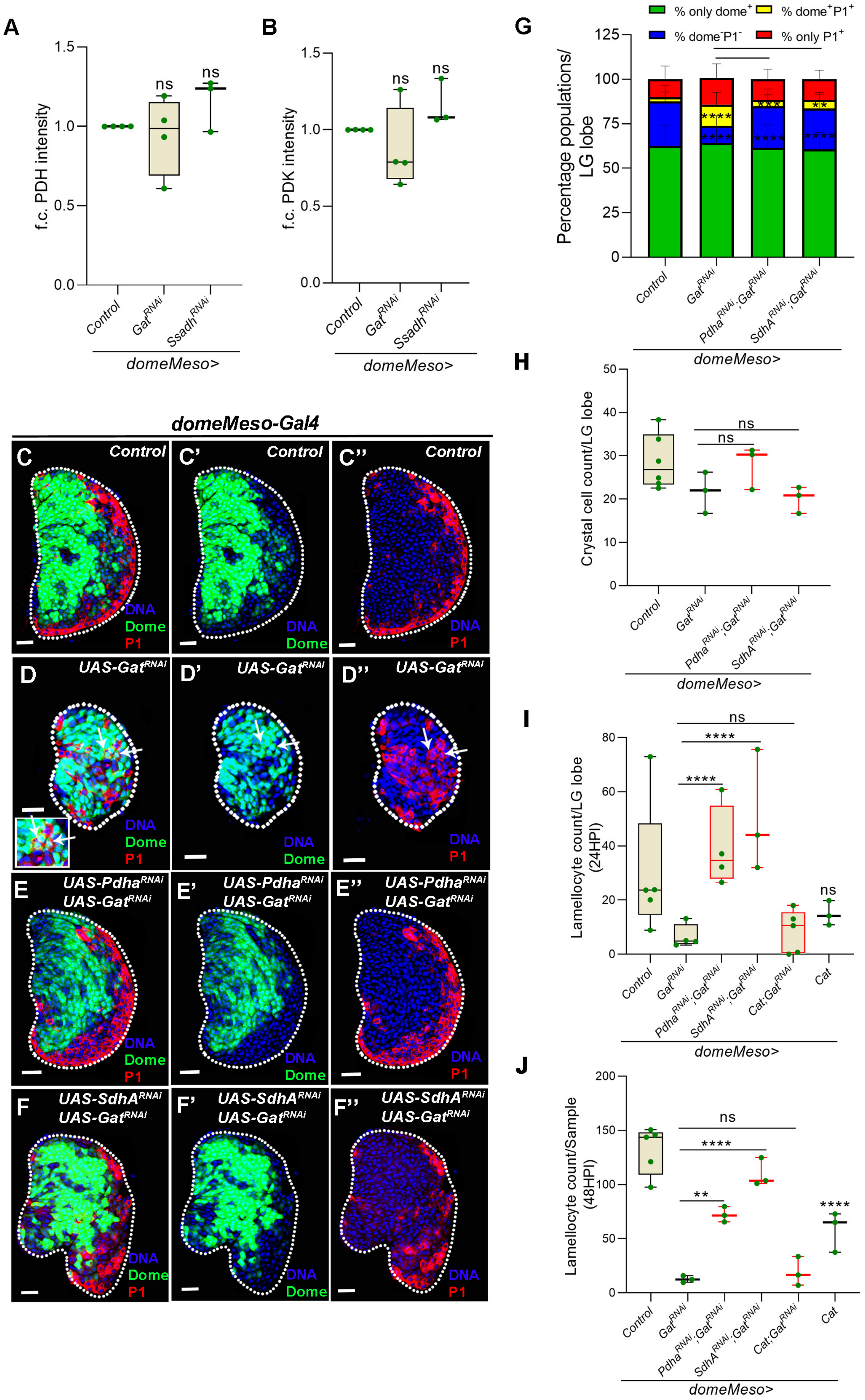

Development • Supplementary information

Fig. S5. GABA catabolism dependent control of TCA activity maintains blood progenitor homeostasis and immune response upon wasp-infection.

(A-B) Relative fold change in lymph gland MZ **(A)** PDH levels in *domeMeso>GFP*/+ (control, N=4, n=40), *domeMeso>GFP*/*Gat*^{RNAi} (N=4, n=33, p=0.6817), and *domeMeso>GFP*/*Ssadh*^{RNAi} (N=3, n=30, p=0.1197) and **(B)** PDK levels in *domeMeso>GFP*/+ (control, N=4, n=47), *domeMeso>GFP*/*Gat*^{RNAi} (N=4, n=41, p=0.1101), and *domeMeso>GFP*/*Ssadh*^{RNAi} (N=3, n=30, p=0.3347).

(C-F") Representative images showing lymph gland growth and differentiation status, **(C-C")** control (*domeMeso-Gal4,UAS-GFP*/+) **(C)** dome$^+$ (green) and P1$^+$ (red), **(C')** dome$^+$(green) and **(C'')** P1$^+$ (red), **(D-D")** expressing *Gat*^{RNAi} in progenitor cells (*domeMeso-Gal4,UAS-GFP;UAS-Gat*^{RNAi}) leads to small lymph gland size and appearance of **(D)** dome$^+$p1$^+$ overlap population (shown in inset and by white arrows) along with an increase in **(D")** P1 population, expressing **(E-E")** *Pdha*^{RNAi} in *Gat*^{RNAi} (*domeMeso-Gal4,UAS-GFP;UAS-Pdha*^{RNAi}*;UAS-Gat*^{RNAi}) and **(F-F")** *SdhA*^{RNAi} in *Gat*^{RNAi} (*domeMeso-Gal4,UAS-GFP;UAS-SdhA*^{RNAi}*;UAS-Gat*^{RNAi}) rescues the lymph gland growth and differentiation defect of **(D-D")** *Gat*^{RNAi}. For quantifications, refer to **G**.

(G) Quantifications of lymph gland differentiation status shown as percentage of only dome$^+$(green), dome$^-$P1$^-$(blue), dome$^+$P1$^+$(yellow) and only P1$^+$(red) populations per lymph gland lobe. p-values are presented in the preceding order. *domeMeso>GFP*/+ (control, n=36) and *domeMeso>GFP/Gat*^{RNAi} (n=20, p=0.7812, <0.0001, <0.0001, 0.0889), *domeMeso>GFP/Pdha*^{RNAi}*;Gat*^{RNAi} (n=34, p=0.4635, <0.0001, 0.0005, 0.2920), and *domeMeso>GFP/SdhA*^{RNAi}*;Gat*^{RNAi} (n=31, p=0.2428, <0.0001, 0.0045, 0.3084).

(H) Quantifications of crystal cell count per lymph gland lobe in *domeMeso>GFP*/+ (control, N=6, n=63) and *domeMeso>GFP/Gat*^{RNAi} (N=3, n=20, p=0.8298), *domeMeso>GFP/Pdha*^{RNAi}*;Gat*^{RNAi} (N=3, n=37, p=0.4133), and *domeMeso>GFP/SdhA*^{RNAi}*;Gat*^{RNAi} (N=3, n=32, p=0.9671).

(I) Quantifications of lamellocyte count per lymph gland lobe at 24HPI in *domeMeso>GFP*/+ (control, N=5, n=58) and *domeMeso>GFP/Gat*^{RNAi} (N=4, n=38, p=0.0426), *domeMeso>GFP/Pdha*^{RNAi}*;Gat*^{RNAi} (N=4, n=52, p<0.0001), *domeMeso>GFP/SdhA*^{RNAi}*;Gat*^{RNAi} (N=3, n=28, p<0.0001), *domeMeso>GFP/Cat;Gat*^{RNAi} (N=5, n=32, p=0.9966) and *domeMeso>GFP/Cat* (N=3, n=18, p=0.7201).

Development • Supplementary information

(J) Quantifications of lamellocyte count in circulation at 48HPI in *domeMeso>GFP*/+ (control, N=5, n=49) and *domeMeso>GFP/Gat^{RNAi}* (N=3, n=29, p<0.0001), *domeMeso>GFP/Pdha^{RNAi};Gat^{RNAi}* (N=3, n=26, p=0.0064), *domeMeso>GFP/SdhA^{RNAi};Gat^{RNAi}* (N=3, n=29, p= p<0.0001), *domeMeso>GFP/Cat;Gat^{RNAi}* (N=3, n=36, p=0.9998) and *domeMeso>GFP/Cat* (N=3, n=27, p<0.0001).

Data is presented as median plots (*p<0.05;**p<0.01;***p<0.001,****p<0.0001,n.s.=non-significant), two-way ANOVA, Tukey's multiple comparisons test and Dunnett's multiple comparison test for **G** (mean±SD). f.c.= fold change. MZ=Medullary Zone. Scale bar: 20µm.'n'=lymph gland lobes and number of animals analysed for **J**. 'N'= number of experimental repeats (green dot). DAPI marks DNA. HPI indicates hours post-infection. Comparisons for significance are with control values, unless marked by horizontal lines for other respective comparisons and red bars represent rescue combinations. Lymph gland lobes are outlined with a white border and for clarity purposes the accompanying background containing other tissues, such as ring gland, brain, dorsal vessel, etc., has been removed.

Development • Supplementary information

Fig. S6

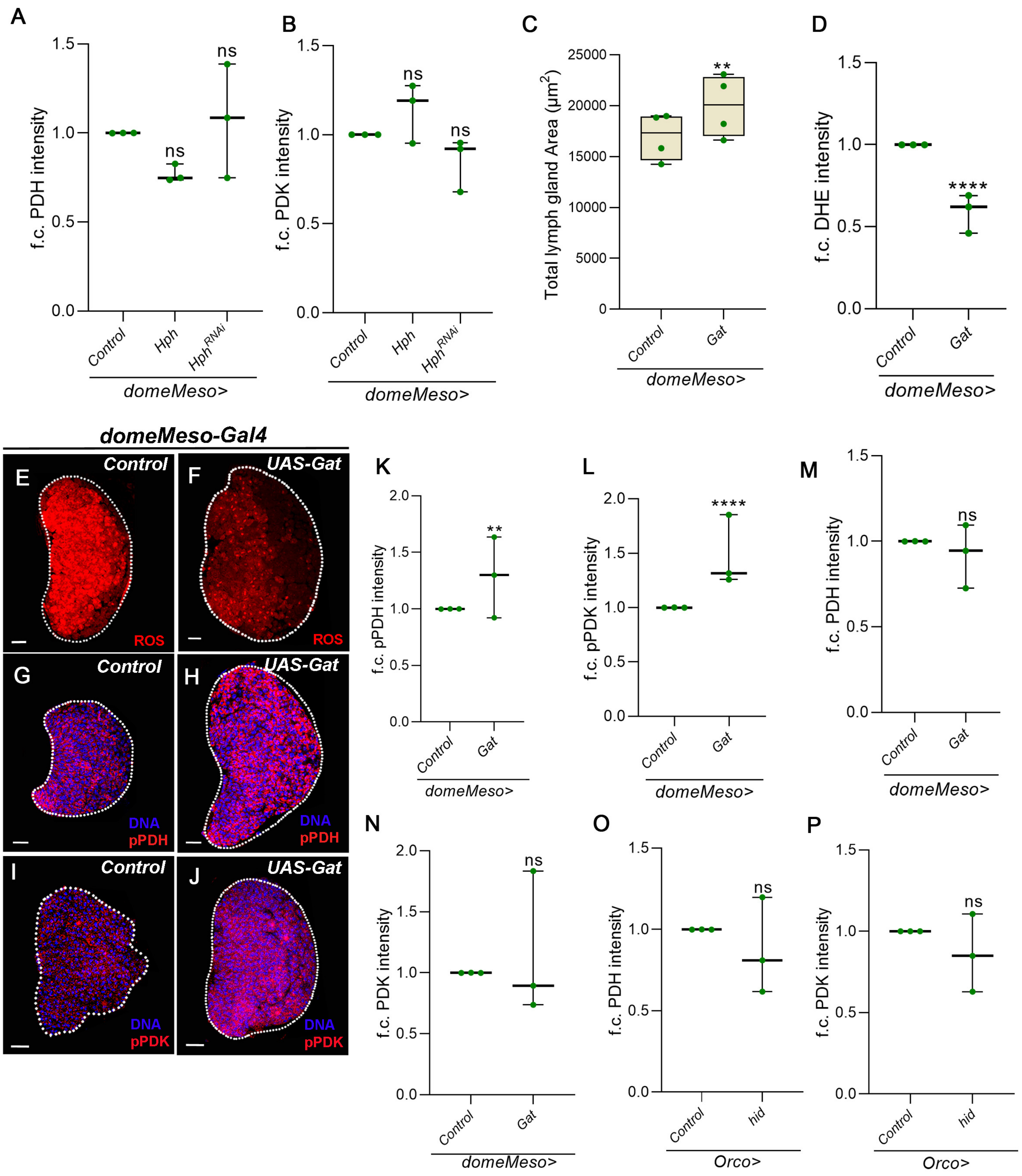

Development • Supplementary information

Fig. S6. Increased GABA uptake increases lymph gland growth and regulates ROS homeostasis.

(A-B) Relative fold change in lymph gland MZ **(A)** PDH levels in *domeMeso>GFP/+* (control, N=3, n=40), *domeMeso>GFP/Hph* (N=3, n=14, p=0.0746), and *domeMeso>GFP/Hph^{RNAi}* (N=3, n=36, p=0.8879) and **(B)** PDK levels in *domeMeso>GFP/+* (control, N=3, n=35), *domeMeso>GFP/Hph* (N=3, n=24, p=0.1651), and *domeMeso>GFP/Hph^{RNAi}* (N=3, n=28, p=0.0705).

(C) Quantification for lymph gland area in *domeMeso>GFP/+* (control, N=4, n=50) and *domeMeso>GFP/Gat* (N=4, n=40, p=0.0023).

(D) Relative fold change in lymph gland ROS (DHE) levels in *domeMeso>GFP/+* (control, N=3, n=34) and *domeMeso>GFP/Gat* (N=3, n=30, p<0.0001).

(E-J) Over-expressing **(F,H,J)** *Gat* in progenitor cells (*domeMeso-Gal4,UAS-GFP;UAS-Gat*) leads to significant reduction in **(F)** ROS levels, increase in **(H)** pPDH and **(J)** pPDK levels as compared to **(E,G,I)** control (*domeMeso-Gal4,UAS-GFP/+*). For quantifications, refer to **D,K,L.**

(K-N), Relative fold change in lymph gland MZ **(K)** pPDH levels in *domeMeso>GFP/+* (control, N=3, n=40) and *domeMeso>GFP/Gat* (N=3, n=31, p=0.0022) **(L)** pPDK levels in *domeMeso>GFP/+* (control, N=3, n=42) and *domeMeso>GFP/Gat* (N=3, n=30, p<0.0001), **(M)** PDH levels in *domeMeso>GFP/+* (control, N=3, n=37) and *domeMeso>GFP/Gat* (N=3, n=17, p=0.4426) and **(N)** PDK levels in *domeMeso>GFP/+* (control, N=3, n=31) and *domeMeso>GFP/Gat* (N=3, n=40, p=0.4377).

(O,P) Relative fold change in lymph gland **(O)** PDH levels in *Orco>/+* (control, N=3, n=24) and *Orco>/Hid* (N=3, n=33, p=0.9983) and **(P)** PDK levels in *Orco>/+* (control, N=3, n=34) and *Orco>/Hid* (N=3, n=32, p=0.1166).

Data is presented as median plots (*p<0.05;**p<0.01;***p<0.001,****p<0.0001,n.s.=non-significant), two-way ANOVA, Tukey's multiple comparisons test. f.c.= fold change. MZ=Medullary Zone. Scale bar: 20µm.'n'=lymph gland lobes. 'N'= number of experimental repeats (green dot). DAPI marks DNA. Lymph gland lobes are outlined with a white border and for clarity purposes the accompanying background containing other tissues, such as ring gland, brain, dorsal vessel, etc., has been removed.

Development • Supplementary information
