# Supplementary material for: Blood progenitor redox homeostasis through olfaction-derived systemic GABA in hematopoietic growth control in Drosophila
Source: Development. 2021 Dec 1;149(8):dev199550. doi: 10.1242/dev.199550 (PMC8733872; doi:10.1242/dev.199550)
Supplement: Reviewer comments [file develop-149-199550_review_history.pdf]

Blood progenitor redox homeostasis through olfaction-derived systemic GABA in hematopoietic growth control in *Drosophila*

Manisha Goyal, Ajay Tomar, Sukanya Madhwal and Tina Mukherjee
DOI: 10.1242/dev.199550

Editor: Irene Miguel-Aliaga

Review timeline
Original submission: 24 February 2021
Editorial decision: 19 April 2021
First revision received: 21 July 2021
Editorial decision: 24 August 2021
Second revision received: 19 September 2021
Accepted: 24 September 2021

Original submission

First decision letter

MS ID#: DEVELOP/2021/199550

MS TITLE: Blood progenitor redox homeostasis through GABA control of TCA cycle in Drosophila hematopoiesis

AUTHORS: Manisha Goyal, Ajay Tomar, Sukanya Madhwal, and Tina Mukherjee

I have now received all the referees' reports on the above manuscript, and have reached a decision. The referees' comments are appended below, or you can access them online: please go to BenchPress and click on the 'Manuscripts with Decisions' queue in the Author Area.

As you will see, the referees express considerable interest in your work, but have some significant criticisms and recommend a substantial revision of your manuscript before we can consider publication. If you are able to revise the manuscript along the lines suggested, which may involve further experiments, I will be happy receive a revised version of the manuscript. Your revised paper will be re-reviewed by one or more of the original referees, and acceptance of your manuscript will depend on your addressing satisfactorily the reviewers' major concerns. Please also note that Development will normally permit only one round of major revision.

We are aware that you may be experiencing disruption to the normal running of your lab that make experimental revisions challenging. If it would be helpful, we encourage you to contact us to discuss your revision in greater detail. Please send us a point-by-point response indicating where you are able to address concerns raised (either experimentally or by changes to the text) and where you will not be able to do so within the normal timeframe of a revision. We will then provide further guidance. Please also note that we are happy to extend revision timeframes as necessary.

Please attend to all of the reviewers' comments and ensure that you clearly highlight all changes made in the revised manuscript. Please avoid using 'Tracked changes' in Word files as these are lost in PDF conversion. I should be grateful if you would also provide a point-by-point response detailing how you have dealt with the points raised by the reviewers in the 'Response to Reviewers' box. If you do not agree with any of their criticisms or suggestions please explain clearly why this is so.

© 2021. Published by The Company of Biologists under the terms of the Creative Commons Attribution License (https://creativecommons.org/licenses/by/4.0/).

Reviewer 1

Advance summary and potential significance to field

This study builds upon the nice Madhwal et al. 2020 eLife paper showing that olfaction modules systemic GABA, which is utilised by blood progenitors via the TCA cycle to generate succinate. Succinate then regulates Hif/Sima activity, thus controlling lamellocyte differentiation. In the new study, Goyal et al. uncover a HIF independent pathway linking the TCA cycle to blood progenitor differentiation-this time involving ROS and PDK. They show that GABA and succinate dehydrogenase activity in progenitors raises ROS and, when this is high enough, it can inhibit lymph gland growth and disturb progenitor maintenance. To prevent this, progenitors catabolize GABA to limit pyruvate and thus carbon entry into the TCA cycle. They go on to show that this limit on anaplerosis involves succinate repression of HIF prolyl hydroxylase (Hph), which helps to maintain PDK activity, thus inhibiting PDH and lowering TCA cycle activity. In turn, this allows glycolysis with low-OXPHOS, which supports normal progenitor development and differentiation.

In summary, this is a very competent and thorough genetic and cellular analysis of an interesting new Sima-independent pathway that, via ROS, can inhibit lymph gland growth. The data are of high quality and many of the findings are well substantiated, although the statistics do need clarifying. On the less positive side, I am left wondering about two major questions. Is the new ROS pathway ever physiologically relevant? And why is an apparently key element of the pathway, Hph, not required? It is essential that the authors address these two questions (elaborated below) and my other major points before publication.

Comments for the author

MAJOR POINTS
1. Is the ROS circuit biologically relevant for the lymph gland?
Several of the manipulations that lower DHE have little/no effect on lymph gland size or on differentiated cell populations. e.g SdhA RNAi. Rather it is only when ROS is made excessively high in artificial conditions that it represses growth. Moreover Gat RNAi plus Hph RNAi has normal levels of DHE but may decrease lymph gland area (Fig 2K,O). It therefore seems that physiological levels of ROS may not play much of a role in regulating lymph gland growth. So can the authors provide evidence that any physiological stimulus encountered by a fly e.g an odor (Madhwal et al. 2020) will lead to a level of ROS high enough to inhibit lymph gland growth. Without this evidence, the worry is that the authors new growth-repressive ROS pathway may not be physiologically relevant.

2. The authors inferred role of Hph, which is central to their conclusions and model does not appear to be supported by the data.
"We prospose that nhibition of Hph activity, by GABA limited excessive ROS levels in progenitor cells, whose accumulation retarded lymph gland growth" (bottom of page 7). If the authors model is really correct then Dome-MESO>Hph[rnai] on its own should increase ROS, yet it fails to change DHE levels. Moreover, Dome-MESO>Hph[rnai] has no significant effect on lymph gland area (Figure 1K). Also, it was previously reported in Madhwal et al. 2020 that Hph[rnai] "leads to comparable number of lamellocytes in lymph gland". Thus, the Hph RNAi results are not compatible with the authors interpretations and models in this manuscript (Figures 1A and 6M).

3. Related point. The fact that Hph/fatiga overexpression does have a phenotype (whereas RNAi alone does not) suggests that the authors may be missing the most important prolyl hydroxylase(s). Either the authors should explicitly acknowledge this limitation of their conclusions or provide evidence for which prolyl hydroxylases are required for lymph gland growth and differentiation.

4. Statistical analysis. N numbers are specified (e.g n=10) but I could not see a clear explanation of what this means. Is this 10 individuals, each one from 10 independent experiments done on different days or is it 10 individuals from the same experiment. If it is the latter, then they are not independent biological replicates and additional experiments are required for every graph where this is the case. The authors really should use mixed-effect models not t-tests to account properly for the different variances between, versus within, experiments. This point is particularly critical where the effect sizes are small and a lot rides on the interpretation e.g Figure 2N for Gat RNAi versus Catalase;Gat RNAi.

© 2021. Published by The Company of Biologists under the terms of the Creative Commons Attribution License (https://creativecommons.org/licenses/by/4.0/).

5. The majority of genetic manipualtions use RNAi, which can be subject to off-target effects. The authors nicely show for the PDK RNAi line that it is on target with respect to loss of pPDH but this still leaves open off target effects that could contribute to the ROS and lymph gland size effects. It is therefore important for future proofing this paper that, at least for a few key results such as PDK RNAi, there is confirmation using a second non-overlapping RNAi sequence or a non-RNAi genetic method.

6. Can the authors clarify if succinate is involved in a TCA inhibitory feedback loop i.e succinate from GABA activates PDK to inhibit the TCA cycle, which also makes succinate. The model in Figure 6M is drawn in such a way that this point is very confusing. However, we know that TCA generated succinate can be exported from the mitochondria and so presumably will contribute to the GABA-catabolised pool of succinate.
MINOR POINTS 7. Shim et al 2013 review paper is cited but it is missing from the reference list. Also, it is cited in the Introduction for GABA signalling but I can't find any reference to GABA in the review itself.

8. The clarity of the Abstract could be improved. For example, the statement about how GABA establishes control of Sima is not clearly explained how this works. Also, I'm not sure what "metabolic underpinnings" in the last sentence really means. In the main manuscript there are numerous cases of typos and incorrect grammar that need to be fixed.

9. The images in Fig. 2 A-C and some other cases appear to be too saturated to be accurately quantified. How was ROS intensity quantified in these cases and compared with those of other manipulations?

10. To assess the effect of succinate food properly, the authors should include the effect of succinate on the control genotype (dome-MESO-Gal4>GFP/+) in Figures 2F & 2G.

<u>Reviewer 2</u>

Advance summary and potential significance to field

In eukaryotic cells, pyruvate can be either fermented to lactate or converted to acetyl-coA, and feed the TCA cycle. This binary decision depends largely on the activation of pyruvate dehydrogenase (PDH), which is negatively regulated by PDK-dependent phosphorylation. This binary decision is believed to regulate proliferation and differentiation in many developmental contexts, although concrete examples of cell fate regulation by this mechanism are only starting to emerge. This work highlights the importance of the TCA cycle regulation in growth control of the Drosophila lymph gland and blood cell differentiation. The authors show that succinate generated by GABA intracellular catabolism is a key regulator of PDK activation, and thereby of pyruvate entry into the TCA cycle.
Enhancement of the TCA cycle results in accumulation of Reactive Oxygen Species (ROS) that in turn inhibit lymph gland growth, and impair blood cell differentiation. This study therefore defines a new mechanism for the control of lymph gland growth and blood cell differentiation, which is based on the regulation of pyruvate entry in the TCA cycle.

Comments for the author

In this study Goyal and coworkers analyze how the modulation of Reactive Oxygen Species (ROS) levels by GABA catabolism affect lymph gland growth and blood progenitor differentiation. They found that reduction of GABA intracellular catabolism leads to ROS accumulation, and impairment of lymph gland growth and blood progenitor differentiation. This regulation depends on succinate, the end product of GABA catabolism, as supplementation of succinate to the culture medium, largely reverts the effect. GABA/succinate modulation of ROS levels is mediated by PDK-dependent pyruvate dehydrogenase (PDH) phosphorylation, and entry of pyruvate into the TCA cycle. Inhibition of the TCA cycle can reduce ROS back to normal levels, and restore normal growth and differentiation of blood cells. Contrary to previous reports, ROS reduction in the lymph gland does not affect growth or differentiation in this context. The authors propose that the HIF Prolyl Hydroxylase, which is known to be inhibited by succinate, promotes PDK activation and PDH phosphorylation (inhibition), thereby reducing TCA cycle activity. They provide evidence that this

© 2021. Published by The Company of Biologists under the terms of the Creative Commons Attribution License (https://creativecommons.org/licenses/by/4.0/).

increase of TCA cycle activity, and ROS accumulation is independent of Sima, the alpha subunit of the Hypoxia Inducible Factor (HIF).

Most of the experiments of this study are well controlled and properly performed, and the conclusions are indeed supported by the data. This is especially true for the demonstration that ROS derive from the TCA cycle in the lymph gland, and thus that PDK is a key regulatory switch for ROS generation.

The experiments showing that GABA catabolism is crucial for ROS production through PDK regulation are also convincing and properly performed. While the effect of ROS as inhibitors of lymph gland growth is well-characterized, the analysis of their effect on blood cell differentiation is incomplete. Why do the authors analyze plasmatocyte differentiation only? They should look at crystal cells and lamellocyte markers as well!! It is definitely important to know if the genetic manipulations performed, including those that affect GABA catabolism, PDK/PDH expression or phosphorylation and TCA cycle activity, also affect crystal cells or lamellocyte differentiation. The experiments that support the notion that the HIF Prolyl Hydroxylase is a key modulator of ROS levels by regulating PDK in a HIF/Sima independent manner are not well conceived, poorly controlled and essentially insufficient (see below).

In my opinion, this part of the work should be eliminated from the paper.
Performing all the necessary experiments to demonstrate that the reported effects are mediated by the Prolyl Hydroxylase in a Sima-independent manner would require a complete study on itself, involving many additional experiments that I believe are beyond the scope of this work. As the authors state at the Discussion section, PDK is a well-known transcriptional target of HIF, and thereby HIF and its prolyl hydroxylase regulate pyruvate entry into the TCA cycle, ultimately controlling ROS abundance. The authors also mention in their Discussion that in mammalian myeloid cells, GABA and succinate regulate HIF Prolyl Hydroxylases, and thereby HIFa stabilization, resulting in control of cell differentiation (Tannahill et al, Nature 2013). The evidence that the authors provide that this mechanism does not operate in the Drosophila lymph gland is poor and insufficient. This reviewer needs compelling evidence to accept that PDK is a novel hydroxylation target of the HIF Prolyl Hydroxylase and that all the observed downstream effects are indeed HIF/Sima-independent. I am aware that several studies have claimed that HIF Prolyl Hydroxylases can hydroxylate non HIF targets, although I should stress that these studies have been collectively challenged, and remain controversial (Cockman et al, eLife 2019).

Besides these central considerations, I wish to mention a couple of simple points that have not been properly controlled. 1) When the authors claim that the Hph RNAi can reverse the effect of Gat or Ssadh silencing, they should include a second UAS construct (UAS-GFP.RNAi or even UAS-LacZ) when they express the Gat RNAi alone (Fig 6 A-H). Otherwise, it could well be that the apparent “rescue” is actually due to titration of the available Gal4 in the cell. This applies to several other figures in which similar rescue experiments where attempted. 2) When the authors claim that silencing of the Prolyl hydroxylase have effects that are Sima-independent, they should suppress the expression of both proteins together, and show that the effect is indistinguishable from silencing the prolyl hydroxylase alone. It is quite surprising that they didn’t attempt to do that (Figures 1, 2 and 6). 3) When they overexpress the Prolyl Hydroxylase (Fig. 1G), which of the three isoforms of the hydroxylase do they overexpress? 4) When they express a Sima RNAi, (i.e. Fig 6 I, J) and see no effect on PDH or PDK phosphorylation, how do they know that Sima is properly silenced? There are many ways in which they could have controlled this. 5) When they assess the effect of Hph or Sima silencing on pPDK or pPDH abundance, why not assessing total PDK levels? (and perhaps also total PDH). As mentioned above, it is well established in other biological systems that PDK is an important HIF transcriptional target, and thus the first point to address is as whether total PDK levels are modified after manipulation of HIF or Hph levels.

In any case, as said above, even if these experiments were properly done, I would not be convinced of a Sima-independent effect of the prolyl hydroxylase on PDK activation. This would be “front page” news for biochemists and cell biologists!! An extensive study aimed at demonstrating the alternative hydroxylation target would be required, and in my opinion is clearly beyond the scope of the current work.

© 2021. Published by The Company of Biologists under the terms of the Creative Commons Attribution License (https://creativecommons.org/licenses/by/4.0/).

In summary, I would be happy to consider this manuscript again, if the experiments that involve Sima and its hydroxylase are eliminated, and the above-mentioned experiments related to crystal cells and lamellocytes differentiation are performed.

Minor points:
1- Page 12, and throughout the paper, "…expression levels of pPDK and pPDH…". It is not actually "expression". It could be "accumulation" of pPDK/pPDH or phosphorylation of PDK/PDH.
2- Page 8, "…we assessed expression levels of the key TCA enzymes PDK and PDH". PDK and PDH are not TCA cycle enzymes.
3- Page 11"…its activity in GABA metabolic mutants was the key source of precocious ROS generation…" "precocious" is not correct. "excessive" or "increased" could replace it.
4- Figure 3L Blue reference "P1 negative area", instead of "Negative area".

First revision

Author response to reviewers' comments

Reviewer 1 Advance summary and potential significance to field...

This study builds upon the nice Madhwal et al. 2020 eLife paper showing that olfaction modules systemic GABA, which is utilised by blood progenitors via the TCA cycle to generate succinate. Succinate then regulates Hif/Sima activity, thus controlling lamellocyte differentiation. In the new study, Goyal et al. uncover a HIF independent pathway linking the TCA cycle to blood progenitor differentiation-this time involving ROS and PDK. They show that GABA and succinate dehydrogenase activity in progenitors raises ROS and, when this is high enough, it can inhibit lymph gland growth and disturb progenitor maintenance. To prevent this, progenitors catabolize GABA to limit pyruvate and thus carbon entry into the TCA cycle. They go on to show that this limit on anaplerosis involves succinate repression of HIF prolyl hydroxylase (Hph), which helps to maintain PDK activity, thus inhibiting PDH and lowering TCA cycle activity. In turn, this allows glycolysis with low-OXPHOS, which supports normal progenitor development and differentiation.

In summary, this is a very competent and thorough genetic and cellular analysis of an interesting new Sima-independent pathway that, via ROS, can inhibit lymph gland growth. The data are of high quality and many of the findings are well substantiated, although the statistics do need clarifying. On the less positive side, I am left wondering about two major questions. Is the new ROS pathway ever physiologically relevant? And why is an apparently key element of the pathway, Hph, not required? It is essential that the authors address these two questions (elaborated below) and my other major points before publication.
-We thank the reviewer for the appreciation of our work and also appreciate the important concerns raised. We have made all possible attempts to address every point and strongly feel that with these changes the manuscript has improved in its overall quality and also clearer in terms of the model that is now being proposed. Please find our detailed response to each comment raised in the sections following below.

Reviewer 1 Comments for the author
MAJOR POINTS
1. Is the ROS circuit biologically relevant for the lymph gland?
Several of the manipulations that lower DHE have little/no effect on lymph gland size or on differentiated cell populations. e.g SdhA RNAi. Rather it is only when ROS is made excessively high in artificial conditions that it represses growth. Moreover Gat RNAi plus Hph RNAi has normal levels of DHE but may decrease lymph gland area (Fig 2K,O). It therefore seems that physiological levels of ROS may not play much of a role in regulating lymph gland growth. So can the authors provide evidence that any physiological stimulus encountered by a fly e.g an odor (Madhwal et al. 2020) will lead to a level of ROS high enough to inhibit lymph gland growth. Without this evidence, the worry is that the authors new growth-repressive ROS pathway may not be physiologically relevant.

© 2021. Published by The Company of Biologists under the terms of the Creative Commons Attribution License (https://creativecommons.org/licenses/by/4.0/).

-As pointed out by the reviewer, we also agree that our data do not support any role for ROS at its physiological levels in regulating lymph gland growth. This is evident in conditions where down-regulating ROS levels either via blocking TCA cycle components (PdhaRNAi, SdhARNAi, Fig. 3O and Supp. Fig. 4A-C) or by over-expressing ROS scavengers (UAS-Catalase, Fig. 2N, Q), did not change lymph gland size. On the contrary, raising the levels of ROS, such as in condition with increased TCA activity (PdkRNAi, Fig. 3O and Supp. Fig. 3J, 4D) or with the down-regulation of ROS scavenging proteins (CatalaseRNAi, Sod2RNAi, Fig. 2L, M, Q and Fig. S2H), the growth of the lymph gland was abrogated. In GatRNAi condition, we observed an increase in lymph gland ROS levels (Fig. 2B, G) and a corresponding reduction in lymph gland size (Fig. 1C, H). The size was restored by either via down-regulation of TCA components (Fig. 4X and Supp. Fig. 5C-F), or over-expression of Catalase (Fig. 2O-Q) as a means to scavenge the excessive ROS and independently with loss of Hph in GatRNAi condition (Fig. 5P). These data do suggest that while progenitor cells maintain ROS in them for functions that remain unclear at the current moment (and is currently being investigated in the lab), the homeostasis of ROS in progenitor cells was necessary to accommodate lymph gland growth. As any further increase over and above this basal threshold prevented lymph gland growth.

Hence the question of physiology that would lead to any increase in ROS and affect lymph gland development is a very valuable one. As suggested by the reviewer, we undertook experiments to assess physiological stimuli that would be relevant to moderate ROS in this tissue. We undertook the assessment of odors and olfaction on this ROS pathway. We observed that animals that are unable to smell show an increase in lymph gland ROS levels (Fig. 6C, D, G) and are smaller in size (Fig. 6B). Genetic means to ablate olfactory receptor neurons led to a striking increase in lymph gland ROS levels (Fig. 6C, D, G). In this genetic context a reduction in progenitor pPDH (Fig. 6E, H, J) and pPDK levels (Fig. 6F, I, K) was also apparent. This data suggested a role for odor sensing in moderating progenitor TCA activity and subsequently ROS levels. These data with loss of olfaction leading to increased ROS and lymph gland growth defect implied that as long as animals were smelling environmental odors, the ROS in the hematopoietic system was kept under check. We propose that subsequent to activation of olfactory signaling, the downstream cascade leading to the release of neuronal GABA (as shown in our previous studies (Madhwal et al., 2020; Shim et al., 2013) leading to its internalization and metabolism by blood-progenitor cells limited TCA activity and moderated excessive ROS production. Thereby allowing lymph glands to grow and achieve their final size. We are thankful to the reviewer for suggesting this experiment. These data indeed bring forth a physiological context to the ROS pathway which was missing in our previous version.

Given that in our previous work (Madhwal et al., 2020) we have also shown the influence of pathogenic wasp-odors which via elevating systemic GABA and subsequently progenitor GABA metabolism enabled superior immune responses, we also tested the influence of pathogenic wasp-odors (WOF) on lymph gland growth. Interestingly, we find that prior exposure to pathogenic odors (WOF condition) led to increase in lymph gland size than seen in regular conditions (Fig. 6O). In WOF condition, we also observed that progenitor ROS was lower (Fig. 6L, P) and the levels of TCA activity was reduced (Fig. 6M, N, Q, R), than their corresponding controls raised in regular food environment. The increased lymph gland size phenotype was also seen when progenitors over-expressed GABA-transporter (Gat) (Supp. Fig. 6G), as a means to internalize more GABA or in conditions when controls were fed with succinate (Fig. 1H) (as a means to increase the GABA catabolic end-product). In all these conditions, we also observed reduction in progenitor ROS (Fig. 2D, G for succinate fed controls and Supp. Fig. 6H, I, J for Gat over-expression) and TCA activity (Fig. 4U, V for Succinate fed controls and Supp. Fig. 6K-P for Gat over-expression) than their corresponding controls. While the data further validate the influence of odors and GABA catabolism being necessary and sufficient in moderating lymph gland sizes, the mechanism by which GABA promotes lymph gland growth is unlikely to be a consequence of down-regulating TCA/ROS levels (Fig. 2Q, 3O). Thus, the factors driving growth downstream of GABA metabolism are independent of ROS/TCA and remain to be investigated.

2. The authors inferred role of Hph, which is central to their conclusions and model does not appear to be supported by the data.
“We prospose that inhibition of Hph activity, by GABA limited excessive ROS levels in progenitor cells, whose accumulation retarded lymph gland growth” (bottom of page 7). If the authors model is really correct then Dome-MESO > Hph[rnai] on its own should increase ROS, yet it fails to change DHE levels. Moreover, Dome-MESO>Hph[rnai] has no significant effect on lymph gland area (Figure 1K). Also, it was previously reported in Madhwal et al. 2020 that Hph[rnai] “leads to comparable

© 2021. Published by The Company of Biologists under the terms of the Creative Commons Attribution License (https://creativecommons.org/licenses/by/4.0/).

number of lamellocytes in lymph gland”. Thus, the Hph RNAi results are not compatible with the authors interpretations and models in this manuscript (Figures 1A and 6M).
-We understand the point raised by the reviewer and agree that Hph data unlike GABA metabolism is not entirely supportive of its necessity in controlling lymph gland growth in homeostasis. We have re-done Hph manipulations to carefully assess the involvement of Hph in lymph gland growth and ROS modulation (Fig. 5).

From our previous work we know, GABA metabolism derived succinate inhibits Hph function which leads to stabilization of Sima protein in blood cells which drives immune response (Madhwal et al., 2020). Hence in the absence of GABA, the reduction in succinate leads to elevation in Hph activity which causes reduction in Sima protein leading to abrogation in lamellocyte response. On the contrary, increase in GABA metabolism or succinate levels, or inhibition of Hph activity promotes Sima protein expression in blood-progenitor cells and consequently leads to increased immune response. Hence, GABA loss and gain of function data are phenocopied by Hph gain and loss of function data.

However, in the growth context, while GABA gain of function led to increased LG growth and reduction in TCA/ROS levels, loss of Hph function failed to show any increase in lymph gland size and remained comparable to controls (Fig. 5P). The readout of TCA activity (pPDH level) showed no change (Fig. 5H, I, R, S) and ROS remained unaffected (Fig. 5G, Q). On the other hand, like GABA loss of function, where reduction in lymph gland size (Fig. 1H), the gain in Hph function recapitulated these phenotypes (Fig. 5A-F, P-S). Not only a reduction in lymph gland size was noticed (Fig. 5P), over-expression of Hph also led to elevation in TCA activity (Fig. 5E, F, R, S) and subsequently ROS levels (Fig. 5D, Q). Based on this data, we hypothesized that in the absence of GABA metabolism and succinate, an increase in Hph activity leads to elevated TCA activity and ROS levels in progenitor cells that causes the size reduction seen in GatRNAi. As a result when HphRNAi was co-expressed in progenitor cells expressing GatRNAi animals, a significant recovery of lymph gland size (Fig. 5P) , ROS (Fig. 5J in comparison to M and U), pPDH (Fig. 5K in comparison to N and R) and pPDK levels (Fig. 5L in comparison to O and S) was noticed. These data implied that Hph function was not contributing toward lymph gland growth in homeostasis. However, in the absence of GABA, the growth defect could be due to increased Hph activity. These data suggested that like TCA activity and ROS levels, gain of Hph function over and above the basal threshold, was sufficient to down-regulate PDK function leading to increased TCA activity and precocious ROS generation which retarded lymph gland growth. Any further reduction in Hph level than its basal level in homeostasis did not contribute to promote growth (Fig. 5P). Thus, suggestive of Hph independent means of growth control via GABA. As is also mentioned in the subsequent comment about other Hph isoforms (Acevedo et al., 2010) and any redundancy in their functions, this is a very likely possibility and our current data is limited in this regard.
Hence, we have now presented the involvement of Hph with respect to these data. We have made the involvement of Hph more clearer in the text and in the model as well. We thank the reviewer, as these points have made us interpret our data with more rigor and present a better model (Fig. 7).

3. Related point. The fact that Hph/fatiga overexpression does have a phenotype (whereas RNAi alone does not) suggests that the authors may be missing the most important prolyl hydroxylase(s). Either the authors should explicitly acknowledge this limitation of their conclusions or provide evidence for which prolyl hydroxylases are required for lymph gland growth and differentiation.
-This is an excellent point and we acknowledge that the discrepancies between the gain and loss of function data with Hph could be indicative of involvement of other prolyl hydroxylases and needs validation. Unfortunately, we did contact the Wappner laboratory to obtain reagents to modulate the other Fga isoforms (Acevedo et al., 2010), but were unable to receive them due to the current pandemic and shipping restrictions. We mention this limitation in the text and present our Hph data in light of these possibilities.

4. Statistical analysis. N numbers are specified (e.g n=10) but I could not see a clear explanation of what this means. Is this 10 individuals, each one from 10 independent experiments done on different days or is it 10 individuals from the same experiment. If it is the latter, then they are not independent biological replicates and additional experiments are required for every graph where this is the case. The authors really should use mixed-effect models not t-tests to account properly for the different variances between, versus within, experiments. This point is particularly critical

© 2021. Published by The Company of Biologists under the terms of the Creative Commons Attribution License (https://creativecommons.org/licenses/by/4.0/).

where the effect sizes are small and a lot rides on the interpretation e.g Figure 2N for Gat RNAi versus Catalase;Gat RNAi.
-We appreciate this point. We have re-done the statistical analysis with mixed effect models and also added more numbers where ever needed. We now state explicitly in the statistical data analysis (Materials and methods) hat "n" implies the total number of animals analyzed from all experimental repeats. In all cases each experiment has been repeated a minimum 3 times and, in each set, at least 5-10 animals have been analyzed. We have plotted our data as median plots and non-parametric two tailed Mann-Whitney U test is employed for pairwise comparisons and Kruskal-Wallis test-Dunn's multiple comparison test has been utilized for multiple comparisons to account for the variation between and within the experiments. The experiments redone to increase "n" value are the following:

1. increased "n" values for ROS analysis in:
domeMeso > GatRNAi(on RF, SF and NAC supplementation), SsadhRNAi (on RF, SF and NAC supplementation), PdhaRNAi, PdhaRNAi;GatRNAi, PdkRNAi (BL28635 and BL35142), SdhARNAi, SdhARNAi;GatRNAi, UAS-Catalase, Cat;GatRNAi, CatalaseRNAi, UAS-Gat, HphRNAi, HphRNAi;GatRNAi, UAS-Hph, Orco > Hid, control animals on SF and WOF.

2. increased "n" values for lymph gland growth analysis in:
domeMeso > GatRNAi(on RF, SF and NAC supplementation), SsadhRNAi (RF, SF and NAC supplementation), PdhaRNAi, PdhaRNAi;GatRNAi, PdkRNAi (BL28635 and BL35142), SdhARNAi, SdhARNAi;GatRNAi, UAS-Catalase, Cat;GatRNAi, CatalaseRNAi, Sod2RNAi, UAS-Gat, HphRNAi, HphRNAi;GatRNAi, UAS-Hph, Orco > Hid, Control animals on SF and WOF.

3. increased "n" values for staining for pPDH, pPDK, PDH and PDK:
domeMeso > GatRNAi(RF and SF), SsadhRNAi (RF and SF), PdhaRNAi (pPDH and PDH), PdkRNAi(pPDH and PDH), UAS-Gat, HphRNAi, HphRNAi;GatRNAi (pPDH and pPDK), UAS-Hph, Orco > Hid, Control animals on SF and WOF.

4. Differentiation analysis for plasmatocytes (P1), crystal cells (PPO1) in homeostasis in : PdhaRNAi, PdkRNAi, SdhARNAi, GatRNAi, PdhaRNAi;GatRNAi, SdhARNAi;GatRNAi and lamellocytes (Myospheroid) post wasp-infection (24HPI, lymph gland and 48HPI, circulation) in: PdhaRNAi, PdkRNAi, SdhARNAi, GatRNAi, PdhaRNAi;GatRNAi, SdhARNAi;GatRNAi , UAS-Catalase and Cat;GatRNAi.

5. The majority of genetic manipualtions use RNAi, which can be subject to off-target effects. The authors nicely show for the PDK RNAi line that it is on target with respect to loss of pPDH but this still leaves open off target effects that could contribute to the ROS and lymph gland size effects. It is therefore important for future proofing this paper that, at least for a few key results such as PDK RNAi, there is confirmation using a second non-overlapping RNAi sequence or a non-RNAi genetic method.
-We had provided data on size reduction using an additional RNAi for Pdk (domeMeso > PdkRNAi (BL35142)) in Suppl. Fig. 3M (now Fig. S3J). We now also provide ROS analysis for this additional line which is shown in Fig. S3I. The data obtained are consistent with both the RNAi lines.

6. Can the authors clarify if succinate is involved in a TCA inhibitory feedback loop i.e succinate from GABA activates PDK to inhibit the TCA cycle, which also makes succinate. The model in Figure 6M is drawn in such a way that this point is very confusing. However, we know that TCA generated succinate can be exported from the mitochondria and so presumably will contribute to the GABA-catabolised pool of succinate.
-Our data support the independence of the GABA derived succinate from the TCA derived succinate. In the absence of GABA metabolism, a reduction in pPDK with a concomitant decrease in pPDH levels is detected, which implies increased TCA activity in these mutants. Importantly, restoring these animals (Gat RNAi and SsadhRNAi conditions) with succinate (via feeding larvae succinate rich food), shown in Fig. 4Q-V, a significant restoration in pPDK levels (Fig. 4R, T in comparison to K, L and V) and consequently, pPDH levels (Fig. 4Q, S in comparison to H, I and U) is detected. Additionally, blocking TCA (via PDH inhibition) (Fig. 3J, K, N, O and Supp. Fig. 4B-B", E-H), which would also result in reduction in succinate generation (TCA derived), did not recapitulate GABA loss of function data. Thus showing that succinate from GABA breakdown is independent of TCA derived

© 2021. Published by The Company of Biologists under the terms of the Creative Commons Attribution License (https://creativecommons.org/licenses/by/4.0/).

succinate, and functioned specifically to activate PDK as a means to repress PDH function and consequently TCA activity.

The independency of GABA and TCA-derived succinate are indicative of independent pools of succinate in a cell that perform different roles. Our previous work (Madhwal et al., 2020), also implied similar results where GABA breakdown derived succinate was necessary to mount immune response, while blocking the steps of the TCA that also generated succinate did not seem to be necessary. Thus, it appears that distinct pools of succinate generated from different metabolic reactions serve independent roles. How this is manifested and regulated are questions that need to be asked and remain to be explored. We state this in the text and have edited the model (Fig. 7) to represent this aspect with better clarity. We thank the reviewer for bringing this point.

MINOR POINTS
7. Shim et al 2013 review paper is cited but it is missing fro2m the reference list. Also, it is cited in the Introduction for GABA signalling but I can't find any reference to GABA in the review itself.
-This is now done. We apologize for this error.

8. The clarity of the Abstract could be improved. For example, the statement about how GABA establishes control of Sima is not clearly explained how this works. Also, I'm not sure what "metabolic underpinnings" in the last sentence really means. In the main manuscript there are numerous cases of typos and incorrect grammar that need to be fixed.
-We have now modified the abstract and corrected the text for all the grammatical and typos.

9. The images in Fig. 2 A-C and some other cases appear to be too saturated to be accurately quantified. How was ROS intensity quantified in these cases and compared with those of other manipulations?
-The medullary zone containing the blood progenitor cells have elevated ROS than the differentiating cells located at the outer layer of the lymph gland called the cortical zone (Owusu-Ansah & Banerjee, 2009). The image acquisition settings are chosen to best capture this difference in control lymph glands without causing saturation in majority of the pixels. However, at times due to variations in ROS levels some images can look saturated, which is perhaps the reason why the images shown in Fig. 2A-C appeared so. This image acquisition settings chosen to capture control images is thereafter kept constant for all other genotypes conducted within the same experimental batch. The ROS quantifications of these images is done by measuring mean fluorescence intensity which is represented as fold change of mean intensities with respect to the corresponding batch control. This is now mentioned in the methods section. The heavily saturated images have been replaced with better representative images of the data. We thank the reviewer for raising this point.

10. To assess the effect of succinate food properly, the authors should include the effect of succinate on the control genotype (dome-MESO-Gal4 > GFP/+) in Figures 2F & 2G.
- This is now done. We have raised control animals on succinate supplemented diet and find that these animals show lower ROS in progenitor cells (Fig. 2D, G) and have a larger lymph glands (Fig. 1E, H). These animals also have higher levels of pPDK, pPDH (Fig. 4U, V). These control data are provided in Figures 1E, 1H, 2D, 2G, 4U and 4V.

Reviewer 2 Advance summary and potential significance to field
In eukaryotic cells, pyruvate can be either fermented to lactate or converted to acetyl-coA, and feed the TCA cycle. This binary decision depends largely on the activation of pyruvate dehydrogenase (PDH), which is negatively regulated by PDK-dependent phosphorylation. This binary decision is believed to regulate proliferation and differentiation in many developmental contexts, although concrete examples of cell fate regulation by this mechanism are only starting to emerge. This work highlights the importance of the TCA cycle regulation in growth control of the Drosophila lymph gland and blood cell differentiation. The authors show that succinate generated by GABA intracellular catabolism is a key regulator of PDK activation, and thereby of pyruvate entry into the TCA cycle.
Enhancement of the TCA cycle results in accumulation of Reactive Oxygen Species (ROS) that in turn inhibit lymph gland growth, and impair blood cell differentiation. This study therefore defines a new mechanism for the control of lymph gland growth and blood cell differentiation, which is based on the regulation of pyruvate entry in the TCA cycle.

© 2021. Published by The Company of Biologists under the terms of the Creative Commons Attribution License (https://creativecommons.org/licenses/by/4.0/).

-We thank the reviewer for the much appreciation of the work and also the constructive critique that was provided. The comments have been very useful in drafting the revised version and we have made all possible attempts to address every concern raised. We feel that with these new changes, either through the introduction of new data or through removal of data as suggested by the reviewer, the revised version is much improved in terms of its overall quality and scientific content and expect that the current draft will be acceptable.

Reviewer 2 Comments for the author
In this study Goyal and coworkers analyze how the modulation of Reactive Oxygen Species (ROS) levels by GABA catabolism affect lymph gland growth and blood progenitor differentiation. They found that reduction of GABA intracellular catabolism leads to ROS accumulation, and impairment of lymph gland growth and blood progenitor differentiation. This regulation depends on succinate, the end product of GABA catabolism, as supplementation of succinate to the culture medium, largely reverts the effect. GABA/succinate modulation of ROS levels is mediated by PDK-dependent pyruvate dehydrogenase (PDH) phosphorylation, and entry of pyruvate into the TCA cycle. Inhibition of the TCA cycle can reduce ROS back to normal levels, and restore normal growth and differentiation of blood cells. Contrary to previous reports, ROS reduction in the lymph gland does not affect growth or differentiation in this context. The authors propose that the HIF Prolyl Hydroxylase, which is known to be inhibited by succinate, promotes PDK activation and PDH phosphorylation (inhibition), thereby reducing TCA cycle activity. They provide evidence that this increase of TCA cycle activity, and ROS accumulation is independent of Sima, the alpha subunit of the Hypoxia Inducible Factor (HIF).

Most of the experiments of this study are well controlled and properly performed, and the conclusions are indeed supported by the data. This is especially true for the demonstration that ROS derive from the TCA cycle in the lymph gland, and thus that PDK is a key regulatory switch for ROS generation. The experiments showing that GABA catabolism is crucial for ROS production through PDK regulation are also convincing and properly performed.

While the effect of ROS as inhibitors of lymph gland growth is well-characterized, the analysis of their effect on blood cell differentiation is incomplete. Why do the authors analyze plasmatocyte differentiation only? They should look at crystal cells and lamellocyte markers as well!! It is definitely important to know if the genetic manipulations performed, including those that affect GABA catabolism, PDK/PDH expression or phosphorylation and TCA cycle activity, also affect crystal cells or lamellocyte differentiation.
-We thank the reviewer for this valuable point. We have now done a thorough analysis of differentiation of all cell lineages. The data on crystal cells and lamellocyte differentiation are shown in the following Figures:

1. Crystal cell counts for domeMeso > GatRNAi and SsadhRNAi have been shown previously in (Madhwal et al., 2020) and their numbers do not change. We have now done crystal cell analysis in mutants affecting Pdha, Pdk expression and TCA cycle activity (SdhA) mutants. While down-regulation of TCA activity in blood progenitors do not show any significant change in crystal cell counts, increase in TCA activity by PdkRNAi leads to a dramatic defect in crystal cell formation as seen for the other cell types. Crystal cell data for domeMeso > PdhaRNAi, SdhARNAi and PdkRNAi is shown in Supp. Fig. 4F, rescue backgrounds with GatRNAi, PdhaRNAi;GatRNAi and SdhARNAi;GatRNAi is shown in Supp. Fig. 5H, where no defect was seen.

2. Lamellocytes have been quantified as done previously (Madhwal et al., 2020) at 24HPI for lymph gland (Supp. Fig. 4G and Supp. Fig. 5I) and at 48HPI for circulation lamellocyte count (Supp. Fig. 4H and Supp. Fig. 5J).
The following are the new data with respect to lamellocytes that have been added:
-Lymph gland lamellocytes (24HPI): domeMeso > PdhaRNAi, SdhARNAi, PdkRNAi, GatRNAi (Fig. S4G), GatRNAi, PdhaRNAi;GatRNAi, SdhARNAi;GatRNAi, Catalase, Catalase;GatRNAi (Fig. S5I).
-Circulation lamellocytes (48HPI): domeMeso > PdhaRNAi, SdhARNAi, PdkRNAi, GatRNAi (Fig. S4H), GatRNAi , PdhaRNAi;GatRNAi, SdhARNAi;GatRNAi, Catalase, Catalase;GatRNAi (Fig. S5J).
We find that loss of Pdha, loss of SdhA (reduced TCA) leads to increased formation of lamellocytes (Supp. Fig. Fig. 4G, H), while reduction in Pdk function (increased TCA) leads to reduction in lamellocyte formation (Supp. Fig. 4G, H). The data show that down-regulation of TCA activity leads to increased lamellocyte induction while excessive TCA activity inhibits it. We also assesses the

© 2021. Published by The Company of Biologists under the terms of the Creative Commons Attribution License (https://creativecommons.org/licenses/by/4.0/).

implications of modulating TCA in GABA pathway mutants where a dramatic defect in lamellocyte formation has been reported in our previous work (Madhwal et al., 2020). Interestingly, we observed that blocking TCA activity in Gat mutant significantly restored the lamellocyte formation defect (Supp. Fig. 5I, J). This data is intriguing and implies GABA-metabolism/TCA cross talk in dictating lamellocyte formation. What remains unclear is how this interaction controls Sima/Hifa function which is central to lamellocyte formation and remains to be investigated. Intrigued by the rescue in lamellocyte formation seen in GatRNAi with reduction in progenitor cell TCA activity, we also assessed if high ROS was involved in moderating this. For this we did the post-infection lamellocyte analysis in UAS-Catalase;GatRNAi condition. Surprisingly, this genetic combination did not restore lamellocyte formation (Supp. Fig. 5I, J) and implied a role for TCA but not ROS in immune response.
We thank the reviewer for this valuable suggestion as these data bring forth the importance of modulating TCA activity in mounting a successful immune response and provides a new dimension for GABA metabolism in regulating TCA activity in moderating infection response. As a part of our future investigations, we will explore this and hope to obtain the mechanistic basis of this process.

The experiments that support the notion that the HIF Prolyl Hydroxylase is a key modulator of ROS levels by regulating PDK in a HIF/Sima independent manner are not well conceived, poorly controlled and essentially insufficient (see below).
In my opinion, this part of the work should be eliminated from the paper. Performing all the necessary experiments to demonstrate that the reported effects are mediated by the Prolyl Hydroxylase in a Sima-independent manner would require a complete study on itself, involving many additional experiments that I believe are beyond the scope of this work. As the authors state at the Discussion section, PDK is a well-known transcriptional target of HIF, and thereby HIF and its prolyl hydroxylase regulate pyruvate entry into the TCA cycle, ultimately controlling ROS abundance. The authors also mention in their Discussion that in mammalian myeloid cells, GABA and succinate regulate HIF Prolyl Hydroxylases, and thereby HIFa stabilization, resulting in control of cell differentiation (Tannahill et al, Nature 2013). The evidence that the authors provide that this mechanism does not operate in the Drosophila lymph gland is poor and insufficient. This reviewer needs compelling evidence to accept that PDK is a novel hydroxylation target of the HIF Prolyl Hydroxylase, and that all the observed downstream effects are indeed HIF/Sima-independent. I am aware that several studies have claimed that HIF Prolyl Hydroxylases can hydroxylate non HIF targets, although I should stress that these studies have been collectively challenged, and remain controversial (Cockman et al, eLife 2019).

-We understand the point raised by the reviewer and also agree with it. The experiments to prove Sima independence of Hph function in PDK regulation will be beyond the scope of the current study. Therefore, as suggested we have now removed all Hif data and toned down our results with Hph function down-stream of GABA metabolism. Additionally, any understanding of the different Hph isoforms and redundancy in their functions during blood development is missing and our current data is also limited in this regard. Hence, we present the involvement of Hph in light of these limitation and in the model (Fig. 7). We sincerely thank the reviewer for suggesting these changes.

Besides these central considerations, I wish to mention a couple of simple points that have not been properly controlled. 1) When the authors claim that the Hph RNAi can reverse the effect of Gat or Ssadh silencing, they should include a second UAS construct (UAS-GFP.RNAi or even UAS-LacZ) when they express the Gat RNAi alone (Fig 6 A-H). Otherwise, it could well be that the apparent “rescue” is actually due to titration of the available Gal4 in the cell. This applies to several other figures in which similar rescue experiments where attempted.
-The point of Gal4 dilution is well taken and we would like to mention that in all our genetic backgrounds driven with domeMeso-Gal4 we have a UAS-GFP transgene that is also expressed under the same Gal4. Thus to address the concern raised on titration of the available Gal4 we now provide quantification of GFP intensities of the rescue genotypes, where multiple UAS’ are driven. This data is shown in Supp. Fig. 2J, which shows no change in GFP intensities and remains comparable across all genetic contexts. This further strengthens the rescues detected and also improves the rigor in our analysis.

2) When the authors claim that silencing of the Prolyl hydroxylase have effects that are Sima-independent, they should suppress the expression of both proteins together, and show that the

© 2021. Published by The Company of Biologists under the terms of the Creative Commons Attribution License (https://creativecommons.org/licenses/by/4.0/).

effect is indistinguishable from silencing the prolyl hydroxylase alone. It is quite surprising that they didn't attempt to do that (Figures 1, 2 and 6).
-Although we have removed the data on Sima from this draft, we will undertake these experiments as they raise a very valuable point that needs to be clarified. This will be taken up as our future endeavour.

3) When they overexpress the Prolyl Hydroxylase (Fig. 1G), which of the three isoforms of the hydroxylase do they overexpress?
- Over-expression construct used in the study expresses the full-length Hph (Frei & Edgar, 2004). The details on Hph isoform, their expression or function during blood progenitor development is not clear and remains to be investigated.

4) When they express a Sima RNAi, (i.e. Fig 6 I, J) and see no effect on PDH or PDK phosphorylation, how do they know that Sima is properly silenced? There are many ways in which they could have controlled this.
-Although this data is no longer a part of the revised version, in our previous manuscript (Madhwal et al., 2020), we have undertaken analysis of the SimaRNAi and have observed almost 50% reduction of Sima protein with this RNAi. But given the conflicting Sima and Hph results in growth, we will undertake experiments that down-regulate Sima function and follow it up with more stringent analysis.

5) When they assess the effect of Hph or Sima silencing on pPDK or pPDH abundance, why not assessing total PDK levels? (and perhaps also total PDH). As mentioned above, it is well established in other biological systems that PDK is an important HIF transcriptional target, and thus the first point to address is as whether total PDK levels are modified after manipulation of HIF or Hph levels.
-We have now done total PDH and PDK expression analysis in UAS-Hph and HphRNAi condition. Both the gain and loss of Hph did not reveal any change in total PDH (Supp. Fig. 6A) or PDK levels (Supp. Fig. 6 B). pPDH and pPDK levels were assessed in Hph modulations. Here, we observed a significant reduction of both pPDH and pPDK in Hph over-expression context (Fig. 5E, F, R, S). This implied that although total levels of these proteins did not change, increased Hph expression could lead to loss of active pPDK levels (Fig. 5F, S) and subsequently pPDH (Fig. 5E, R). In HphRNAi condition, a significant increase in pPDK level was noticed (Fig. 5I, S) but pPDH remained unchanged (Fig. 5H, R). These data are not consistent with Hph via Hif mediated transcriptional regulation of PDK and reflect a complex role for Hph in PDK regulation in homeostasis. This may stem from the fact that there are different isoforms of Hph that work by modulating Sima or independent of Sima. Hence, at this point it is not entirely clear as to how Hph and Sima function in PDK regulation. This needs more thorough investigation and will be taken up in our future studies.

However, based on our data with GABA loss of function, such as in conditions of GatRNAi, which is perhaps a more sensitized background, the loss of succinate resulting in elevated Hph activity leading to loss of PDK activity is seen. This is consistent with gain of Hph function data. Hence the subsequent reduction in Hph expression in GatRNAi being sufficient to restore growth (Fig. 5P), TCA activity (Fig. 5K, L ,in comparison to N, O and R, S) and ROS (Fig. 5J in comparison to M, Q), implicates regulation of Hph function via GABA to keep its activity under check. Over and above this threshold may lead to down-regulation of PDK activity. Our data are however limited in the context of how Hph functions given the lack of understanding of which isoforms are affected and how redundantly they could perform. Hence we present our data now in light of these limitations and have also re-worked our model to project this aspect.

In any case, as said above, even if these experiments were properly done, I would not be convinced of a Sima-independent effect of the prolyl hydroxylase on PDK activation. This would be "front page" news for biochemists and cell biologists!! An extensive study aimed at demonstrating the alternative hydroxylation target would be required, and in my opinion is clearly beyond the scope of the current work.

In summary, I would be happy to consider this manuscript again, if the experiments that involve Sima and its hydroxylase are eliminated, and the above-mentioned experiments related to crystal cells and lamellocytes differentiation are performed.
-We have made all the necessary changes as suggested by the reviewer. While entire data on Sima has been removed, we present the Hph data in light of the limitations about the different isoforms

© 2021. Published by The Company of Biologists under the terms of the Creative Commons Attribution License (https://creativecommons.org/licenses/by/4.0/).

and their functional roles not being clear. We hope this will be acceptable, as it presents a plausible mechanism that operates in the absence of GABA leading to the growth defect. We will be happy to remove this part, in case this representation of the data isn't clear. In addition to this the whole new section on differentiation of different cell lineages has been added which is indeed valuable as it implicates a previously uncharacterized role for TCA in immune response and also opens a new avenue for our investigations.

Minor points:
1-Page 12, and throughout the paper, "…expression levels of pPDK and pPDH…". It is not actually "expression". It could be "accumulation" of pPDK/Ppdh or phosphorylation of PDK/PDH.
-This is now done and we thank the reviewer for suggesting this correction.

2-Page 8, "…we assessed expression levels of the key TCA enzymes PDK and PDH". PDK and PDH are not TCA cycle enzymes.
-We have now re-worded them accurately.

3-Page 11"…its activity in GABA metabolic mutants was the key source of precocious ROS generation…" "precocious" is not correct. "excessive" or "increased" could replace it.
-This is now corrected. We apologize for this.

4-Figure 3L Blue reference "P1 negative area", instead of "Negative area".
-This is now corrected.

Reviewer 3rd

Goyal et al

The manuscript under review explores the role of GABA in regulating Drosophila hematopoiesis. It describes a process whereby systemic GABA is internalized and catabolized in blood progenitors to succinate. Succinate inhibits Hph, which would otherwise inhibit PDK and Sima. Both high PDK and Sima activities are required for lymph gland growth/maintenance.

This is a strong, well-constructed manuscript that presents novel findings.

-We thank the Reviewer for the appreciation of our work and the comments provided. They have been very useful in revising the draft and we have made all changes as suggested. We hope the revised draft will now be acceptable. Please find our detailed response to each comment raised in the sections following here with.

My one major concern is that the manuscript presents rescue experiments in an uncontrolled manner. It often compares conditions with one UAS-RNAi to those with two UAS-RNAis. The problem with not controlling for the number of UAS constructs being driven is that the introduction of additional UAS-RNAis can dilute Gal4 and reduce the efficacy of the initial UAS-RNAi knockdown resulting in an apparent, albeit trivial, rescue. As just one example, in Figure 1H and I it shows UAS-Gat RNAi reduces lymph gland area while expressing UAS-Hph RNAi in a UAS-Gat RNAi background restores lymph gland area. This might indeed indicate that GABA inhibits Hph to maintain the lymph gland OR it could be that introducing the UAS-Hph RNAi has diluted out the Gal4 and made UAS-Gat RNAi less efficacious. In order to be certain about the rescue experiments presented throughout, upon which the manuscript bases many of its conclusions, I feel the appropriate controls must be included. For example, the correct comparison in Figure 1 would be UAS-control RNAi, UAS-Gat RNAi to UAS-Hph RNAi, UAS-Gat RNAi.

-The point of Gal4 dilution has also been raised by Reviewer 2 and is well taken. We have taken care of this issue. In all our genetic backgrounds driven with domeMeso-Gal4, we have a UAS-GFP transgene that is also expressed under the same Gal4. Thus to address the concern raised on titration of the available Gal4 we now provide quantifications of GFP intensities of the rescue genotypes, where multiple UAS' are driven. This data is shown in Supplementary Fig. 2J, which shows no change in GFP intensities and remains comparable across all genetic contexts. This further strengthens the rescues detected and has improved the overall rigor in our analysis.

© 2021. Published by The Company of Biologists under the terms of the Creative Commons Attribution License (https://creativecommons.org/licenses/by/4.0/).

Minor Points

1. It is my understanding that normally TCA cycle stands for “tricarboxylic acid” cycle not “the citric acid” cycle as it is in the text. If the authors agree, I suggest using the more conventional definition

–this is now done.

2. On page 5, the manuscript states “Blocking GABA uptake or the penultimate component of the pathway Ldh, in differentiating Hemolectin+ (Hml+) blood cells did not render any growth defect (Supp. Fig. 1 C, D, F and G).” What is the evidence that the knockdown with Hml-Gal is similarly effective as it is with dome-MESO-Gal4 or TepIV-Gal4? Could this be due to differences in knockdown efficacy? Could the Hml-Gal4 driver simply be weaker in these tissues at these stages? The same question applies to the differences observed between the drivers in the Sima knockdowns.

-The data on Sima and Ldh are now removed as suggested by Reviewer 2. However, in response to the lack of growth seen upon expression of GatRNAi in differentiating cells, we feel it is unlikely to be arising due to differences in the strength of the driver. First, the expression analysis of Gat protein shows higher levels in medullary zone progenitor cells than in differentiating cells Supp. Fig. 1C, C’ and D. This supports Gat requirement seen in progenitor cells. Secondly, our on-going independent work shows that down-regulating Gat in differentiating cells using Hml driver, even though doesn't affect lymph gland size, this genetic combination is sufficient to block lymph gland lamellocyte differentiation in response to wasp-infection. We will be happy to provide this data if needed. Hence, the lack of growth phenotype but requirement in immune response with loss of Gat in differentiating cells using Hml-Gal4, suggests differential role for GABA metabolism in Hemolectin population.

3. The first paragraph on page 6, starts with “In progenitor cells, GABA inhibits Hph activity.” It was unclear whether this is a finding of the paper or a known fact as is stated. If this is already known, please include a citation. If not, perhaps this should be the conclusion of the paragraph not the starting sentence.

–This was a finding made in our previous work (Madhwal et al., 2020) and now cited in the text.

4. In Figure 1G, the GFP positive cells look much larger in the Hph overexpression. Is this the case and, if so, why?

- We have now carefully analyzed the UAS-Hph data and do observe an increase in overall cell size. It is known that gain of Hph function can lead to cell growth (Frei & Edgar, 2004), where Hph function is a key mediator of Cyclin D/Cdk4 function. However, if this is true in Hph over-expressing lymph gland progenitor cells needs to be addressed. We thank the reviewer for bringing this point and indeed appreciate the reviewers fine view of our data.

5. On page 8 the manuscript states “Compared to this, levels of pPDH (Fig. 3D, D’) and pPDK (Fig. 3E, E’) was specifically elevated in dome+ progenitor cells when compared to their levels detected in dome- differentiating cells (Fig. 3D- E’).” I found this hard to see. Can this be quantified by measuring average fluorescent intensity in the dome+ vs dome– cells?

-This is now done and provided in Fig. 3F, G, H and I.

There is substantial evidence that succinate can promote ROS generation via reverse electron transport. In the discussion, the authors should consider discussing how this relates to their findings.

-The succinate derived from the GABA catabolic pathway functions to suppress ROS generation, while the succinate derived from the TCA cycle, via SdhA drives ROS generation. If the succinate derived from TCA via RET drives ROS generation is a very likely possibility and in this context the GABA derived succinate via limiting TCA could be preventing RET. We thank the reviewer for raising this point and have written the discussion to incorporate this aspect of succinate and RET.

© 2021. Published by The Company of Biologists under the terms of the Creative Commons Attribution License (https://creativecommons.org/licenses/by/4.0/).

References:
Acevedo, J. M., Centanin, L., Dekanty, A., & Wappner, P. (2010). Oxygen sensing in Drosophila: multiple isoforms of the prolyl hydroxylase fatiga have different capacity to regulate HIFalpha/Sima. PLoS One, 5(8), e12390. https://doi.org/10.1371/journal.pone.0012390

Frei, C., & Edgar, B. A. (2004). Drosophila cyclin D/Cdk4 requires Hif-1 prolyl hydroxylase to drive cell growth. Dev Cell, 6(2), 241-251. https://doi.org/10.1016/s1534-5807(03)00409-x

Madhwal, S., Shin, M., Kapoor, A., Goyal, M., Joshi, M. K., Ur Rehman, P. M., Gor, K., Shim, J., & Mukherjee, T. (2020). Metabolic control of cellular immune-competency by odors in Drosophila. Elife, 9. https://doi.org/10.7554/eLife.60376

Owusu-Ansah, E., & Banerjee, U. (2009). Reactive oxygen species prime Drosophila haematopoietic progenitors for differentiation. Nature, 461(7263), 537-541. https://doi.org/10.1038/nature08313

Shim, J., Mukherjee, T., Mondal, B. C., Liu, T., Young, G. C., Wijewarnasuriya, D. P., & Banerjee, U. (2013). Olfactory control of blood progenitor maintenance. Cell, 155(5), 1141-1153. https://doi.org/10.1016/j.cell.2013.10.032

Second decision letter

MS ID#: DEVELOP/2021/199550

MS TITLE: Blood-progenitor redox homeostasis through olfaction-derived systemic GABA in hematopoietic growth control in Drosophila

AUTHORS: Manisha Goyal, Ajay Tomar, Sukanya Madhwal, and Tina Mukherjee

I have now received all the referees reports on the above manuscript, and have reached a decision. The referees' comments are appended below, or you can access them online: please go to BenchPress and click on the 'Manuscripts with Decisions' queue in the Author Area.

The overall evaluation is positive and we would like to publish a revised manuscript in Development, provided that the referees' comments can be satisfactorily addressed. Please attend to all of the reviewers' comments in your revised manuscript and detail them in your point-by-point response. If you do not agree with any of their criticisms or suggestions explain clearly why this is so.

I have listed the relevant points below - none of them should require additional experiments.

Replace Or83b in the model Fig. 7 with “olfactory neurons”¤ (rev 1).
Provide the data for individualexperiments for the WOF data (rev 1)
Amend the wording around Hph and generally improve the readabilityof the manuscript (rev 2).
Discuss the subcellular compartmentalization of succinate in their manuscript (rev 3).

Regarding the stats point raised by reviewer 1. It seems to me that the analysis you say you have applied in your response is appropriate as long as you have included independent experiments as one of your variables (i.e. a batch effect). Please clarify whether this is the case and state more clearly in the manuscript which variables you have taken into consideration in your analysis for each of the experiments involving multiple comparisons.

Reviewer 1

Advance summary and potential significance to field

© 2021. Published by The Company of Biologists under the terms of the Creative Commons Attribution License (https://creativecommons.org/licenses/by/4.0/).

This is a very competent and thorough genetic and cellular analysis of an interesting new olfactory neuron-dependent pathway that, via ROS, can inhibit lymph gland growth. The data are of high quality and most of the findings are well substantiated.

Comments for the author

Goyal et al. have now satisfactorily addressed the majority of my major and minor criticisms, some of which necessitated generating new experimental data. They have now dealt adequately with the lack of a requirement for Hph in lymph gland growth, and also most other parameters measured. The inclusion of the new data on the physiological regulation of GABA in lymph gland growth by olfactory neurons is a welcome addition. In particular, the data indicating that Orco-expressing cells, likely olfactory neurons, are required to suppress high ROS and to facilitate lymph gland growth do strengthen the manuscript. These cell ablation data convincingly implicate a role for olfactory neurons but, in the absence of tests with Orco/Or83b mutants, it is overinterpretation to add “Or83b” to the model in Fig. 7 and so this should be removed (a suggestion would be to replace it with “olfactory neurons”).

In contrast to the Orco>hid data, the data for pathogenic wasp odours (WOF, Fig6O-6R), look to have small effect sizes and lower levels of statistical significance. This make me nervous about whether or not the authors conclusions will turn out to be robust. Therefore, it would be much better show all three or more independent experiments separately (Materials and Methods states at least 3 experiments were completed) for these data and thus provide a convincing case that the WOF effects are reproducible between experiments. Alternatively, at this point, it might be a safer publication strategy, in terms of future proofing the conclusions of the paper, to delete these WOF data and, instead, to speculate on this point in the Discussion. The inclusion of just the Orco>hid data would, in my opinion, be sufficient to provide the required physiological relevance for this paper.
Only one major criticism was not adequately addressed-the problem with the statistics (Point 4). The authors state in the response letter that they have used mixed-effect models to account for the different variances between, versus within, experiments but they do not appear to have done so. Other methods need to be used e.g a linear mixed model (LMM) in R with experimental manipulations, such as the genotype, as fixed effects and the independent experiment as a random effect. Statistical inference for fixed effects can then be tested using e.g a Wald Chi-Squared test. At present, data from different experiments are lumped together as if it had been one big experiment-this is not good statistical practice.

Reviewer 2

Advance summary and potential significance to field

Already described when I evalueted the original submission

Comments for the author

My points have been properly addressed. Having eliminated the experiments that attempted to support the claim that the effect of succinate is conveyed by the HIF-Prolyl-Hydroxylase in a HIF-independent manner, the manuscript is now significantly improved, and I don’t have further concerns. However, several sentences related to experiments that involve Hph need to be modified to avoid over-interpretation of the data:
1- Throughout the manuscript, as for example in lines 317, 319, 322, 325 330 and 340, the authors state that Hph “activity” is required for the effects that they observe. However, they have not really assessed or manipulated the activity of the enzyme, and do not have any evidence that such activity is necessary for the biological functions under study. Instead of talking about the “activity”, they could simply state that Hph is necessary for… etc. It could well be that an inactive version of the protein can fulfill the same functions.
This issue is critical, and needs to be assessed in the future, along with a better characterization of Hph function in the regulation of bioenergetics metabolism.

© 2021. Published by The Company of Biologists under the terms of the Creative Commons Attribution License (https://creativecommons.org/licenses/by/4.0/).

2- Along the same line, at the Discussion section (lines 404-405), the authors state: “In conditions with low progenitor GABA metabolism, the lack of succinate generation from this pathway, leads to increased Hph activity and loss of PDK…” That is a wild statement!! They should consider to eliminate or soften considerably this statement.
3- Lines 243 and 252: When the authors refer to “immune response”, do they really mean “wasp egg infection”. Please clarify.
4- Overall, the quality of the text needs to be improved, perhaps by asking for the assistance of a colleague, or hiring a professional service. A few examples of writing errors are listed below, but examples of poor text quality are found all throughout the manuscript:
a) Line 69: “…which is THE end-product of glycolysis…”
b) Line 102 “…the last and rate-limiting step…”
c) Line 109: “…using A similar RNAi…” or “…using THE SAME RNAi…”
d) Line 200: “…the levels of THE phosphorylated form (pPDH) WERE reduced…”
e) Line 209: “…lowering progenitor ROS BELOW physiological levels did not…”
f) Line 274: “…and implied enhanced TCA…” the word “implied” is repeated in the sentence.
g) Line 325: “…We observed that, INCREASED Hph LEVELS led to…”

Reviewer 3

Advance summary and potential significance to field

See previous review.

Comments for the author

Mukherjee et al have addressed most of my previously raised issues. My main issue was to do with incompletely controlled experiments, specifically the possibility of dilution of Gal4 in their rescue experiments. They have somewhat addressed this concern by quantifying the amounts of GFP from a UAS-GFP transgene present in their experiments. While this somewhat addresses my concern, in my opinion it is a poor substitute for doing the appropriate controls as previously recommended by myself and reviewer 2. Small differences in silencing efficacy can have profound influences on phenotype and it is not clear that such differences would be measurable by quantifying GFP. Everything else has been appropriately addressed.

One final thought is that the authors might want to more clearly articulate and discuss the subcellular compartmentalization of succinate in their manuscript.
They are proposing a somewhat unexpected model where cytosolic succinate derived from the GABA catabolic pathway supresses ROS generation, while mitochondrial succinate promotes ROS generation. The two succinate pools are thought to be in equilibrium via the dicarboxylate carrier SLC25A10 (see Murphy and O’Neill-Cell-2018) so how they act independently of one another remains unclear. This is particularly confusing in their model (Figure 7) as they have cytosolic succinate activating PDK (located in mitochondria) resulting in the inhibition of ROS generation, and TCA generated succinate (also mitochondrial) potentially promoting ROS. How succinate in mitochondria does two opposing functions remains unclear and I think this deserves, at minimum, some further discussion. As does why GABA derived succinate doesn’t enter mitochondria directly through the dicarboxylate carrier SLC25A10 to promote ROS.

Second revision

Author response to reviewers' comments

Response to Editor and Reviewer comments

We thank the editor and all the reviewers for their supportive and constructive feedback and overall very useful comments on our manuscript. We have now addressed all remaining concerns

© 2021. Published by The Company of Biologists under the terms of the Creative Commons Attribution License (https://creativecommons.org/licenses/by/4.0/).

with respect to statistical analysis, removal of certain data parts (WOF), and addition of discussion elements on succinate, as suggested by the reviewers.
I address all comments in the point-by-point response herewith.

Comments from the Editor

Dear Dr. Mukherjee,

I have now received all the referees reports on the above manuscript, and have reached a decision. The referees' comments are appended below, or you can access them online: please go to BenchPress and click on the 'Manuscripts with Decisions' queue in the Author Area.

The overall evaluation is positive and we would like to publish a revised manuscript in Development, provided that the referees' comments can be satisfactorily addressed. Please attend to all of the reviewers' comments in your revised manuscript and detail them in your point-by-point response. If you do not agree with any of their criticisms or suggestions explain clearly why this is so.

I have listed the relevant points below - none of them should require additional experiments.

Replace Or83b in the model Fig. 7 with “olfactory neurons” (rev 1).
-We have changed this to ORNs (olfactory receptor neurons).

Provide the data for individual experiments for the WOF data (rev 1)
-We have removed the WOF data, as suggested by Reviewer 1.

Amend the wording around Hph and generally improve the readability of the manuscript (rev 2).
-This is now addressed.

Discuss the subcellular compartmentalization of succinate in their manuscript (rev 3).
-A description on subcellular succinate compartmentalization is also added in the discussion section.

Regarding the stats point raised by reviewer 1. It seems to me that the analysis you say you have applied in your response is appropriate as long as you have included independent experiments as one of your variables (i.e. a batch effect). Please clarify whether this is the case and state more clearly in the manuscript which variables you have taken into consideration in your analysis for each of the experiments involving multiple comparisons.

-We have now redone the analysis to take the batch effect into consideration. Every data is now analysed with two-way ANOVA and Tukey's multiple comparison test with batch as one of the variables. Data from three or more independent experimental repeats were assessed and subsequently graphs were plotted as column graphs. ‘n’ is the total number of animals analysed from all experimental repeats and “N” is the number of experimental repeats and each dot in the graphs represents the number of independent experimental repeats. We have clearly stated this in the methods section and also in the accompanying figure legends.

Reviewer 1 Advance summary and potential significance to field
This is a very competent and thorough genetic and cellular analysis of an interesting new olfactory neuron-dependent pathway that, via ROS, can inhibit lymph gland growth. The data are of high quality and most of the findings are well substantiated.

Reviewer 1 Comments for the author
Goyal et al. have now satisfactorily addressed the majority of my major and minor criticisms, some of which necessitated generating new experimental data. They have now dealt adequately with the lack of a requirement for Hph in lymph gland growth, and also most other parameters measured. The inclusion of the new data on the physiological regulation of GABA in lymph gland growth by olfactory neurons is a welcome addition. In particular, the data indicating that Orco-expressing cells, likely olfactory neurons, are required to suppress high ROS and to facilitate lymph gland growth do strengthen the manuscript. These cell ablation data convincingly implicate a role for

© 2021. Published by The Company of Biologists under the terms of the Creative Commons Attribution License (https://creativecommons.org/licenses/by/4.0/).

olfactory neurons but, in the absence of tests with Orco/Or83b mutants, it is overinterpretation to add “Or83b” to the model in Fig. 7 and so this should be removed (a suggestion would be to replace it with “olfactory neurons”).

-We thank the reviewer for the overall positive review of our work. The incorporation of olfaction data has tremendously enhanced the conceptual context of the work and provides a physiological perspective which was previously missing. As suggested, we have now replaced Or83b in the model with ORNs (olfactory neurons).

In contrast to the Orco > hid data, the data for pathogenic wasp odours (WOF, Fig6O-6R), look to have small effect sizes and lower levels of statistical significance. This make me nervous about whether or not the authors conclusions will turn out to be robust. Therefore, it would be much better show all three or more independent experiments separately (Materials and Methods states at least 3 experiments were completed) for these data and thus provide a convincing case that the WOF effects are reproducible between experiments. Alternatively, at this point, it might be a safer publication strategy, in terms of future proofing the conclusions of the paper, to delete these WOF data and, instead, to speculate on this point in the Discussion. The inclusion of just the Orco > hid data would, in my opinion, be sufficient to provide the required physiological relevance for this paper.

-We agree with the reviewer and have now removed the WOF data for simplification.

Only one major criticism was not adequately addressed-the problem with the statistics (Point 4). The authors state in the response letter that they have used mixed-effect models to account for the different variances between, versus within, experiments but they do not appear to have done so. Other methods need to be used e.g a linear mixed model (LMM) in R with experimental manipulations, such as the genotype, as fixed effects and the independent experiment as a random effect. Statistical inference for fixed effects can then be tested using e.g a Wald Chi-Squared test. At present, data from different experiments are lumped together as if it had been one big experiment-this is not good statistical practice.

-We have now redone the analysis to include the batch effect. Every individual experimental repeat (5-10 animals per experiment) is taken into consideration separately and two-way ANOVA with Tukey’s post-hoc test is applied for multiple comparisons on the data sets. ‘n’ is the total number of animals analysed from all experimental repeats and “N” is the number of experimental repeats and each dot in the graphs represents the number of independent experimental repeats. The overall conclusions from these analyses remain unchanged. Moreover, we appreciate the suggestion, as we acknowledge that the overall quality of the results is now further improved, both in terms of rigor in the analysis and the strength of the conclusions drawn. We sincerely thank the reviewer for bringing this point, which we clearly understood in this round of revision. The detail of our statistical analysis is stated in the Material and Methods section and each figure legend as well. We hope with these changes, the overall stats will be acceptable.

Reviewer 2 Advance summary and potential significance to field
Already described when I evalueted the original submission

Reviewer 2 Comments for the author
My points have been properly addressed. Having eliminated the experiments that attempted to support the claim that the effect of succinate is conveyed by the HIF-Prolyl-Hydroxylase in a HIF-independent manner, the manuscript is now significantly improved, and I don’t have further concerns. However, several sentences related to experiments that involve Hph need to be modified to avoid over-interpretation of the data:

We thank Reviewer 2 for the encouraging remarks on our work. We have now corrected the draft for the remaining text edits and hope with these changes the draft will be acceptable.

1-Throughout the manuscript, as for example in lines 317, 319, 322, 325, 330 and 340, the authors state that Hph “activity” is required for the effects that they observe. However, they have not really assessed or manipulated the activity of the enzyme, and do not have any evidence that such activity is necessary for the biological functions under study. Instead of talking about the

© 2021. Published by The Company of Biologists under the terms of the Creative Commons Attribution License (https://creativecommons.org/licenses/by/4.0/).

"activity", they could simply state that Hph is necessary for… etc. It could well be that an inactive version of the protein can fulfill the same functions.
This issue is critical, and needs to be assessed in the future, along with a better characterization of Hph function in the regulation of bioenergetics metabolism.

-This is now corrected.

2-Along the same line, at the Discussion section (lines 404-405), the authors state: "In conditions with low progenitor GABA metabolism, the lack of succinate generation from this pathway, leads to increased Hph activity and loss of PDK…" That is a wild statement!! They should consider to eliminate or soften considerably this statement.

-This is now corrected.

3-Lines 243 and 252: When the authors refer to "immune response", do they really mean "wasp egg infection". Please clarify.

-This is now updated in the current version.

4-Overall, the quality of the text needs to be improved, perhaps by asking for the assistance of a colleague, or hiring a professional service. A few examples of writing errors are listed below, but examples of poor text quality are found all throughout the manuscript:

a)Line 69: "…which is THE end-product of glycolysis…"
b)Line 102 "…the last and rate-limiting step…"
c)Line 109: "…using A similar RNAi…" or "…using THE SAME RNAi…"
d)Line 200: "…the levels of THE phosphorylated form (pPDH) WERE reduced…"
e)Line 209: "…lowering progenitor ROS BELOW physiological levels did not…"
f)Line 274: "…and implied enhanced TCA…" the word "implied" is repeated in the sentence.
g)Line 325: "…We observed that, INCREASED Hph LEVELS led to…"

-Manuscript is now improved in reading and for writing errors.

Reviewer 3 Advance summary and potential significance to field
See previous review.

Reviewer 3 Comments for the author
Mukherjee et al have addressed most of my previously raised issues. My main issue was to do with incompletely controlled experiments, specifically the possibility of dilution of Gal4 in their rescue experiments. They have somewhat addressed this concern by quantifying the amounts of GFP from a UAS-GFP transgene present in their experiments. While this somewhat addresses my concern, in my opinion it is a poor substitute for doing the appropriate controls as previously recommended by myself and reviewer 2. Small differences in silencing efficacy can have profound influences on phenotype and it is not clear that such differences would be measurable by quantifying GFP. Everything else has been appropriately addressed.

-We thank the reviewer for the support of our work. We also understand the point on Gal4 dilution being made. We will try to address this in our subsequent manuscripts more thoroughly, by using the UAS-RNAi constructs that are now available. Unfortunately, due to the COVID pandemic, our fly shipments were often stuck and due to consistent delays, we received them dead on arrival, which affected these experiments. Hence, we used the GFP quantification as the means to address this concern. Although this may not be absolutely accurate in gauging effects of Gal4 dilution, it should reveal if any major changes in Gal4 activity were the case. In our future endeavours, we will however address this concern rigorously using the RNAi tools and sincerely appreciate the reviewer for this valuable suggestion.

One final thought is that the authors might want to more clearly articulate and discuss the subcellular compartmentalization of succinate in their manuscript.

© 2021. Published by The Company of Biologists under the terms of the Creative Commons Attribution License (https://creativecommons.org/licenses/by/4.0/).

They are proposing a somewhat unexpected model where cytosolic succinate derived from the GABA catabolic pathway supresses ROS generation, while mitochondrial succinate promotes ROS generation. The two succinate pools are thought to be in equilibrium via the dicarboxylate carrier SLC25A10 (see Murphy and O'Neill-Cell-2018) so how they act independently of one another remains unclear. This is particularly confusing in their model (Figure 7) as they have cytosolic succinate activating PDK (located in mitochondria) resulting in the inhibition of ROS generation, and TCA generated succinate (also mitochondrial) potentially promoting ROS. How succinate in mitochondria does two opposing functions remains unclear and I think this deserves, at minimum, some further discussion. As does why GABA derived succinate doesn't enter mitochondria directly through the dicarboxylate carrier SLC25A10 to promote ROS.

-This is now discussed explicitly in the discussion section of the revised draft. Succinate derived from GABA catabolism also constitutes the mitochondrial pool. The opposing roles of these different pools of succinate from GABA catabolism and TCA cycle in ROS regulation is indeed intriguing. But the detailed mechanism of this regulation remains unclear and our speculation in this regard is now presented in the discussion section.

Third decision letter

MS ID#: DEVELOP/2021/199550

MS TITLE: Blood-progenitor redox homeostasis through olfaction-derived systemic GABA in hematopoietic growth control in Drosophila

AUTHORS: Manisha Goyal, Ajay Tomar, Sukanya Madhwal, and Tina Mukherjee
ARTICLE TYPE: Research Article

I am happy to tell you that your manuscript has been accepted for publication in Development, pending our standard ethics checks.

© 2021. Published by The Company of Biologists under the terms of the Creative Commons Attribution License (https://creativecommons.org/licenses/by/4.0/).
